# Dioscin in liver diseases: pharmacological mechanisms, translational potential and future perspectives

**DOI:** 10.3389/fphar.2026.1919617

**Published:** 2026-08-11

**Authors:** Jia Liu, Rulan Sun, Jie Huang, Cheng Wang

**Affiliations:** 1 Department of Pharmacy, West China Hospital, Sichuan University, Chengdu, China; 2 School of Clinical Medical, Chengdu Medical College, Chengdu, China; 3 Department of Pharmacy, The First Affiliated Hospital of Chengdu Medical College, Chengdu, China; 4 Department of Respiratory and Critical Care Medicine, The First Affiliated Hospital of Chengdu Medical College, Chengdu, China

**Keywords:** dioscin, drug delivery systems, hepatoprotection, liver disease, pharmacokinetics, toxicity

## Abstract

Dioscin (C_45_H_72_O_16_, DIO) is a naturally occurring steroidal saponin widely distributed in medicinal plants of the Dioscoreaceae, Liliaceae, and Fabaceae families. Given its remarkable hepatoprotective properties, DIO has attracted increasing attention in recent years. Based on literature retrieved from Web of Science, PubMed, ScienceDirect, and CNKI, this review systematically summarizes the pharmacological activities, molecular mechanisms, toxicological characteristics, and pharmacokinetic properties of DIO in liver diseases. Available evidence demonstrates that DIO exhibits significant therapeutic potential against various liver disorders, including liver injury, metabolic dysfunction-associated steatotic liver disease, cholestatic liver disease, liver fibrosis, and hepatocellular carcinoma. Mechanistically, DIO exerts hepatoprotective effects by regulating oxidative stress, inflammation, lipid metabolism, bile acid homeostasis, apoptosis, autophagy, and ferroptosis through multiple signaling pathways, including Keap1/Nrf2, TGF-β1/Smad, PI3K/Akt/mTOR, Wnt/β-catenin, TLR4/MyD88/NF-κB, and SLC7A11/GPX4. Toxicological studies indicate that DIO is generally well tolerated at therapeutic doses, whereas prolonged exposure or high-dose administration may induce hepatotoxicity, mild hematological toxicity, and gastrointestinal toxicity. Pharmacokinetic investigations reveal that DIO displays slow absorption, extremely low oral bioavailability, and prolonged intestinal retention. After absorption, it is distributed mainly to the liver and lungs, and undergoes deglycosylation, oxidation, and glucuronidation, with fecal excretion serving as the primary elimination route. In addition, this review covers the botanical sources, biological characteristics, novel drug delivery systems, and current research challenges associated with DIO. Overall, it provides a comprehensive basis for the further development and clinical translation of DIO in the prevention and treatment of liver diseases.

## Graphical Abstract

**Figure:**
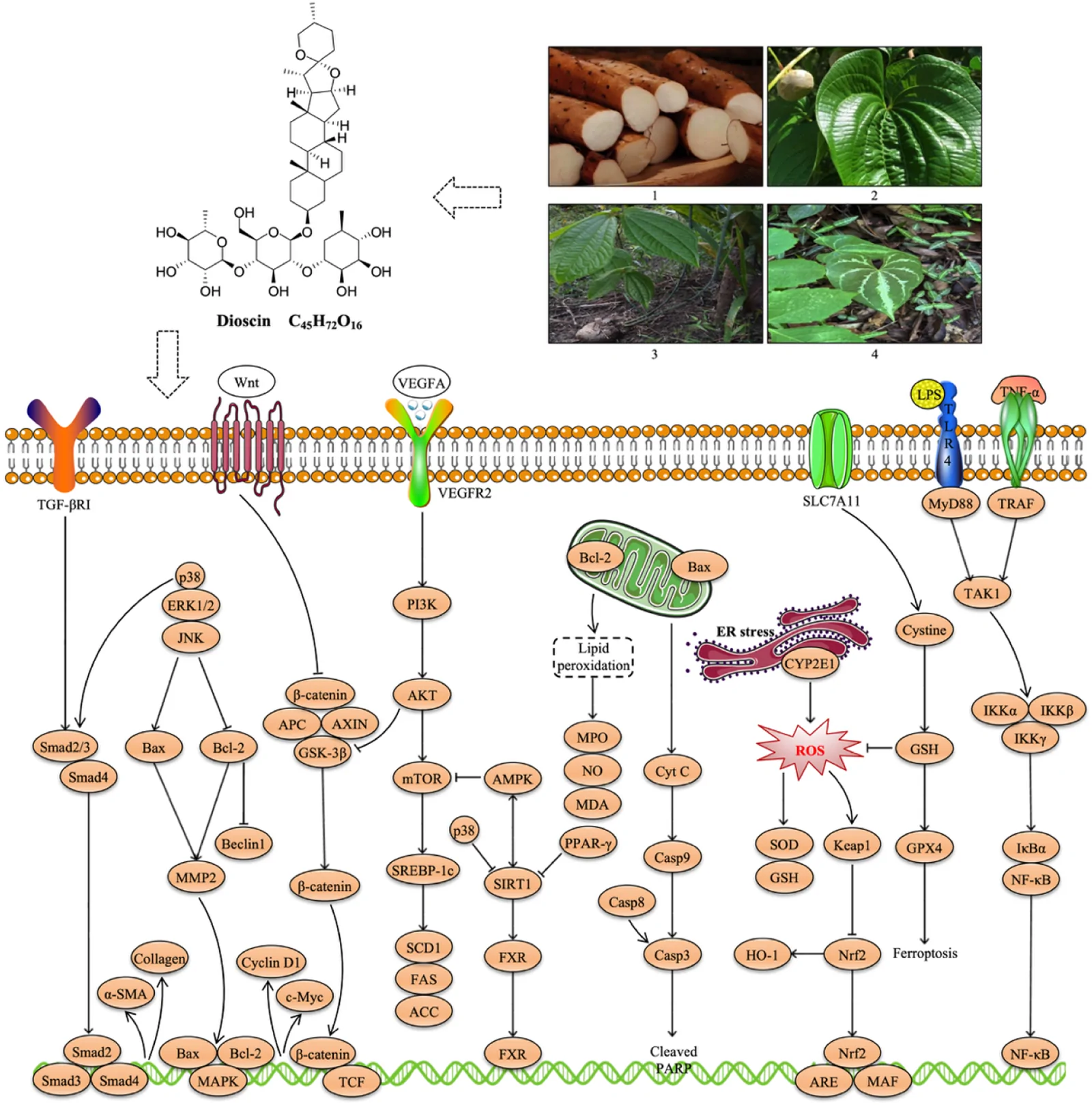

## 1 Introduction

The liver is a vital organ that plays a central role in maintaining metabolic homeostasis, detoxification and biotransformation, immune regulation, and bile secretion. It is also responsible for lipid and glucose metabolism, drug metabolism, and inflammatory responses (Kubes and Jenne, 2018; Zaefarian et al., 2019; Chen et al., 2024). However, liver diseases caused by viral infections, alcohol consumption, metabolic dysfunction, drug toxicity, and immune abnormalities have become major global public health challenges, seriously threatening human health (LeFort et al., 2024; Sanfeliu-Redondo et al., 2024). These conditions encompass a broad spectrum of pathological states, including metabolic dysfunction-associated steatotic liver disease (MASLD), liver fibrosis, cirrhosis, and hepatocellular carcinoma (HCC) (Arroyo et al., 2020; Taru et al., 2024). Although they differ in etiology, their progression frequently involves shared pathological processes such as oxidative stress, inflammatory responses, mitochondrial dysfunction, lipid metabolism disorders, hepatocyte apoptosis, and hepatic stellate cell (HSC) activation (Wang et al., 2021; Kleinert and Horton, 2022; Lai et al., 2024). Despite considerable advances in modern medicine, current therapeutic strategies remain limited by insufficient efficacy, adverse side effects, and a lack of specific targeted drugs (Neshat et al., 2021). Therefore, the development of safe and effective natural compounds with multi-target regulatory properties has emerged as an important direction in the prevention and treatment of liver diseases.

Natural products have attracted increasing attention in the management of liver diseases owing to their wide availability, multi-target activities, and favorable safety profiles (Wang C. et al., 2024; Chen C. et al., 2025; Chen L. et al., 2025; Wang et al., 2026; Wen et al., 2026). Dioscin (DIO), a naturally occurring steroidal saponin primarily isolated from medicinal plants such as *Dioscorea nipponica* and *Dioscorea zingiberensis*, possesses a wide range of pharmacological activities (Tao et al., 2018; Xu et al., 2018). Accumulating evidence has demonstrated that DIO exerts significant hepatoprotective effects in various liver diseases by alleviating lipid accumulation, suppressing oxidative stress and inflammatory responses, inhibiting hepatocyte apoptosis, attenuating liver fibrosis, and suppressing the progression of liver cancer (Lu et al., 2012; Zhang W. et al., 2016; Xu et al., 2017; Song et al., 2019; Yanar et al., 2025). Mechanistically, these protective effects are closely associated with the regulation of multiple signaling pathways, including ERα/AMPK, Sirt1/Nrf2, TLR4/MyD88/NF-κB, PI3K/Akt, MAPK, and autophagy-related pathways (Liu et al., 2015a; Zhang et al., 2015a; Gu et al., 2016; Yao et al., 2016; Xing et al., 2024). In addition, DIO exerts multilayered hepatoprotective effects through improving mitochondrial function and regulating bile acid metabolism (Zhao et al., 2012; Bao et al., 2022). Collectively, these findings suggest that DIO may serve as a promising natural candidate for the prevention and treatment of liver diseases.

In light of the growing research interest in the therapeutic effects of DIO against liver diseases, this review systematically summarizes the pharmacological activities and underlying molecular mechanisms of DIO in various liver disorders, based on literature retrieved from Web of Science, PubMed, ScienceDirect and CNKI databases. Furthermore, this review discusses the pharmacokinetic characteristics, bioavailability, and current challenges associated with the clinical translation of DIO. The aim is to provide a theoretical basis and future perspectives for basic research and clinical application in liver disease therapy.

## 2 Biological characteristics of DIO

**FIGURE 1:**
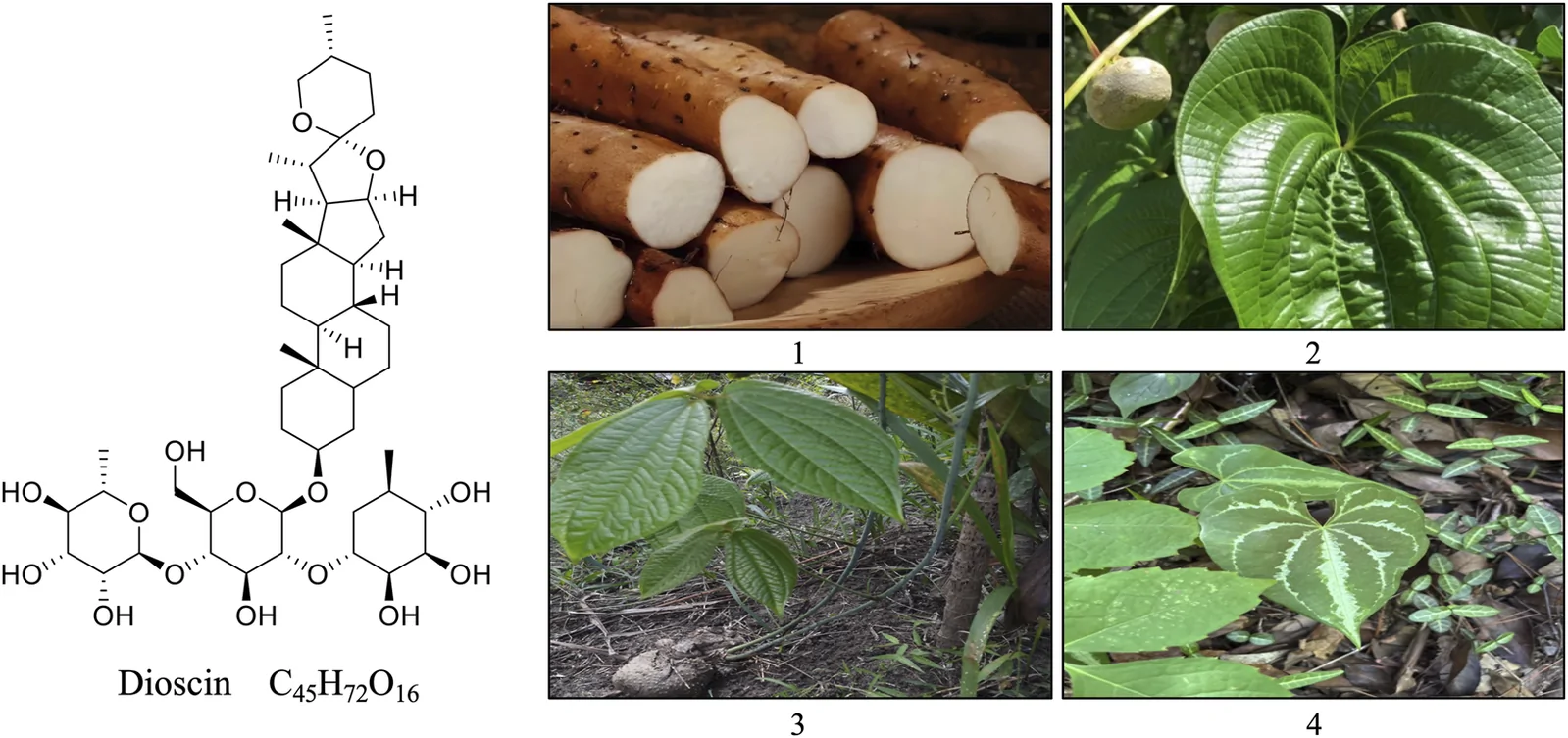
Chemical structure of DIO and its various sources. 1: *Dioscoreae rhizome*; 2: *Dioscorea bulbifera* L.; 3: *Dioscorea hispida* Dennst.; 4: *Dioscorea zingiberensis* C.H.Wright. The images of these medicinal plants were obtained from http://www.gbif.org and https://image.baidu.com.

DIO (C_45_H_72_O_16_, Figure 1) is a naturally occurring steroidal saponin widely distributed in medicinal plants belonging to the families Dioscoreaceae, Liliaceae, and Fabaceae, with particularly high abundance in species of the *Dioscorea* genus (Tao et al., 2018; Yang et al., 2019). Its major natural sources include *D. nipponica*, *D. zingiberensis*, and Chinese yam, among other medicinal herbs (Xu et al., 2018). As a principal bioactive constituent of traditional Chinese medicine (TCM), DIO has attracted considerable attention in natural product research and TCM modernization. Extensive studies have demonstrated that DIO exhibits a wide range of pharmacological activities, including anti-inflammatory, antioxidant, antitumor, antifibrotic, lipid-regulatory and immunomodulatory effects (Li X. et al., 2021; Gao et al., 2024; Wang et al., 2025). Consequently, it has shown promising therapeutic potential for liver diseases, cardiovascular disorders and metabolic diseases.

With respect to its physicochemical properties, DIO is a white or off-white amorphous powder with the identifiers CAS 19057-60-4 and UNII 3B95U4OLWV. Its molecular formula is C_45_H_72_O_16_, with a molecular weight of 869.06. Owing to the presence of both the steroidal aglycone core and multiple sugar moieties, DIO exhibits amphiphilic characteristics, possessing both hydrophilic and lipophilic properties, although its overall aqueous solubility remains relatively poor. It is readily soluble in organic solvents such as methanol, ethanol, and dimethyl sulfoxide, slightly soluble in water, and insoluble in non-polar solvents like petroleum ether. This amphiphilic property enables DIO to interact with cholesterol within cellular membranes, thereby influencing membrane permeability and related intracellular signaling pathways. In addition, DIO demonstrates favorable thermal stability and remains relatively stable under conventional storage conditions. However, it may degrade under high temperatures or in strong acidic or alkaline environments.

Structurally, DIO is classified as a typical spirostanol steroidal saponin. Its basic framework consists of the steroidal sapogenin diosgenin, which is linked to multiple sugar residues through glycosidic bonds. The steroidal nucleus possesses a characteristic rigid tetracyclic structure, whereas the sugar chain is commonly composed of monosaccharides such as rhamnose and glucose. The composition, number and linkage patterns of these sugar moieties not only influence the physicochemical properties of DIO, but are also closely associated with its bioavailability and pharmacological activities. The glycosidic bonds in DIO are easily hydrolyzed under acidic conditions or in the presence of glycosidases, and the resulting metabolites such as diosgenin have important biological activities. Furthermore, the abundant hydroxyl groups in the molecule confer strong hydrogen bond donor and acceptor capacities, facilitating interactions with proteins, enzymes, and membrane receptors, thereby participating in the regulation of various physiological and pathological processes.

In summary, DIO is an important natural steroidal saponin characterized by a unique chemical structure and diverse pharmacological activities. Its potent biological activities, relatively favorable safety profile, and stable physicochemical properties highlight its considerable value in natural drug development and the modernization of TCM. With continued advances in pharmacokinetics, structural modification, and novel drug delivery systems, DIO is expected to show greater clinical potential in the prevention and treatment of liver diseases, metabolic disorders, and cancers. Detailed physicochemical parameters are presented in Table 1, with data obtained from the SciFinder, ChemSpider, and Chemical Book databases.

**TABLE 1:** Physical and chemical properties of DIO.

Name	Dioscin
Source	Dioscoreaceae
CAS number	19057-60-4
EINECS number	618-347-7
CB number	CB2768036
UNII number	3B95U4OLWV
MDL Number	MFCD02094174
Compound type	Steroidal saponins
Molecular formula	C_45_H_72_O_16_
Molecular weight	869.06 g/mol
Form	Powder
Color	White
Solubility	Soluble in DMSO, pyridine, hot methanol/ethanol
InChIKey	VNONINPVFQTJOC-MRFMZFTMNA-N
Density	1.39 ± 0.1 g/cm^3^
pKa	12 ± 0.70 (Predicted)
Boiling point	N/A
Melting point	275–277 °C
Flash point	N/A
Refractivity	1.613
Polar surface area	235.68000
LogP	1.24170
Storage conditions	Sealed, away from light, 2 °C–8 °C

## 3 The hepatoprotective effects and mechanisms of DIO in liver injury

Liver injury is a common pathological process in the occurrence and development of various acute and chronic liver diseases, making it an important experimental paradigm to evaluate the hepatoprotective potential of therapeutic agents (Zhang X. et al., 2024). In recent years, increasing evidence has shown that DIO has a significant hepatoprotective effect in various experimental models. DIO does not act through a single target, but coordinately regulates multiple pathological processes such as oxidative stress, inflammatory responses, mitochondrial dysfunction, apoptosis, and metabolic disorders to alleviate liver injury. These pleiotropic pharmacological activities provide a mechanistic basis for its broad-spectrum hepatoprotective efficacy. The following sections summarize the therapeutic effects and underlying molecular mechanisms of DIO in different types of liver injury.

### 3.1 Chemical-induced liver injury

#### 3.1.1 CCl_4_

CCl_4_ is a classical hepatotoxic agent widely used to establish experimental models of acute liver injury (Scholten et al., 2015). The activation of CCl4 by cytochrome P450 enzymes, particularly CYP2E1, produces the highly reactive trichloromethyl. These free radicals trigger lipid peroxidation, oxidative stress, inflammation, and hepatocyte apoptosis and necrosis, eventually leading to severe liver injury (Unsal et al., 2021).

Therefore, the CCl_4_-induced liver injury model has been extensively employed to evaluate the hepatoprotective effects of candidate drugs. Previous studies have demonstrated that DIO exerts significant protective effects against CCl_4_-induced liver injury, and its hepatoprotective efficacy at a dose of 100 mg/kg is comparable to that of 200 mg/kg silymarin (Lu et al., 2011). Specifically, DIO markedly reduces serum levels of alanine aminotransferase (ALT), aspartate aminotransferase (AST), alkaline phosphatase (ALP), and lactate dehydrogenase (LDH), and ameliorates histopathological liver damage (Lu et al., 2011). Moreover, DIO attenuates lipid peroxidation and inflammatory responses by decreasing the levels of pro-inflammatory cytokines, including tumor necrosis factor α (TNF-α) and interleukin-6 (IL-6), indicating its potent antioxidant and anti-inflammatory activities (Lu et al., 2012).

Further mechanistic investigations have revealed that DIO attenuates hepatocellular injury by modulating mitochondrial apoptosis-related signaling pathways (Lu et al., 2012). Specifically, DIO downregulates the expression of Fas/FasL, increases the Bcl-2/Bax ratio, inhibits the release of cytochrome c from mitochondria into the cytoplasm, and suppresses the activation of Caspase-3 and Caspase-8, thereby effectively reducing hepatocyte apoptosis and necrosis (Lu et al., 2012). In addition, DIO regulates the expression of multiple proteins associated with oxidative stress, inflammation, and cellular injury, including ICAM-1, vimentin, prohibitin, HGF, c-MET, GSTA1, and iNOS, thereby contributing to its hepatoprotective effects (Lu et al., 2012). Proteomic analyses have also identified several potential biomarkers, such as HSPA5, ANXA6, IVD, RPS6, Cygb, and NDPK-A, suggesting that DIO may ameliorate acute liver injury via multi-target and multi-pathway mechanisms (Lu et al., 2011).

Furthermore, in order to improve the solubility and oral bioavailability of DIO, Ju et al. (2019) developed a DIO nanosuspension (DIO-NS). The results demonstrated that DIO-NS exhibited favorable stability and *in vitro* dissolution properties, and showed hepatoprotective activity comparable to that of silymarin in a mouse model of CCl_4_-induced acute liver injury, suggesting that nano-formulation strategies may enhance the clinical application potential of DIO (Ju et al., 2019).

#### 3.1.2 Thioacetamide

Thioacetamide (TAA) is a classic hepatotoxic agent. After metabolic activation by the cytochrome P450 enzymes in liver cells, TAA generates highly active metabolites. These metabolites induce excessive accumulation of reactive oxygen species (ROS), resulting in oxidative stress, lipid peroxidation, inflammatory responses, and ultimately hepatocyte necrosis and apoptosis (Ezhilarasan, 2023; Zhang and Xu, 2024). Therefore, the TAA-induced acute liver injury model has been widely used to evaluate the hepatoprotective effects of candidate drugs and their underlying mechanisms. Notably, Zheng et al. (2018) demonstrated that DIO exerted significant protective effects against TAA-induced acute liver injury in both rats and mice. Compared with the model group, DIO markedly reduced serum ALT and AST levels, alleviated histopathological liver damage, increased the levels of antioxidant indicators including glutathione (GSH), glutathione peroxidase (GSH-Px), and superoxide dismutase (SOD), and decreased malondialdehyde (MDA) content, indicating a potent capacity to attenuate oxidative stress-induced injury (Zheng et al., 2018). In addition, DIO reduced the levels of pro-inflammatory cytokines, such as TNF-α and IL-6, while upregulating IL-10, thereby exerting anti-inflammatory effects (Liu et al., 2021).

Further mechanistic investigations indicated that the hepatoprotective effects of DIO may be closely associated with the regulation of the FXR/AMPK signaling pathway (Zheng et al., 2018). DIO significantly upregulated the expression of FXR, p-AMPKα, and Nrf2, which subsequently promoted the expression of antioxidant-related proteins, including HO-1, NQO-1, GCLM, and GST (Zheng et al., 2018). Meanwhile, DIO suppressed the expression of inflammatory mediators and signaling molecules, including NF-κB (p65), ICAM-1, HMGB1, and COX-2, thereby synergistically mitigating TAA-induced oxidative stress and inflammatory responses (Zheng et al., 2018). Furthermore, the hepatoprotective efficacy of high-dose DIO (100 mg/kg) is comparable to that of silymarin, suggesting its promising therapeutic potential for acute chemical-induced liver injury (Liu et al., 2021).

#### 3.1.3 Dimethylnitrosamine

Dimethylnitrosamine (DMN) is a classic chemical hepatotoxin that is metabolized by hepatic cytochrome P450 enzymes to generate highly reactive intermediates. These intermediates induce excessive ROS production, leading to oxidative stress, lipid peroxidation, inflammatory responses, and activation of apoptosis-related signaling pathways, ultimately resulting in acute liver injury (LeFort et al., 2024). Therefore, the DMN-induced acute liver injury model has been widely employed to explore the pathogenesis of chemical-induced liver injury and the hepatoprotective efficacy of candidate agents.

Zhang W. et al. (2016) demonstrated that DIO exerted significant protective effects against DMN-induced acute liver injury. DIO markedly reduced serum ALT and AST levels, increased the levels of antioxidant indicators including SOD, GSH-Px, and GSH, and decreased the levels of MDA, iNOS, and NO, indicating its potent ability to alleviate oxidative stress-induced injury (Zhang W. et al., 2016). In addition, DIO suppressed inflammatory responses through the regulation of the TLR4/MyD88 signaling pathway, as evidenced by the significant downregulation of inflammatory mediators and signaling molecules, including IL-1β, IL-6, TNF-α, IκB, p50, and p65 (Zhang W. et al., 2016).

Mechanistically, DIO promoted the nuclear translocation of Nrf2 and subsequently upregulated the expression of antioxidant-related proteins, including SIRT1, HO-1, NQO1, GST, and GCLM, thereby enhancing cellular antioxidant defense capacity (Zhang W. et al., 2016). Meanwhile, DIO downregulated the expression of apoptosis-related proteins, including FasL, Fas, p53, Bak, and Caspase-3/9, and increased Bcl-2 expression via the suppression of IRF9, thereby inhibiting hepatocyte apoptosis (Zhang W. et al., 2016). Collectively, these findings suggest that DIO ameliorates DMN-induced acute liver injury through coordinated antioxidant, anti-inflammatory, and anti-apoptotic mechanisms.

### 3.2 Drug-induced liver injury

#### 3.2.1 Methotrexate

Methotrexate (MTX) is an antimetabolite agent widely used for the treatment of malignancies and autoimmune diseases (Schmidt et al., 2022). However, long-term or high-dose administration may induce severe hepatotoxicity. Existing studies have demonstrated that MTX promotes excessive generation of ROS, triggering oxidative stress, lipid peroxidation, and mitochondrial dysfunction. These alterations subsequently activate inflammatory and cell injury-related signaling pathways, ultimately resulting in hepatocellular injury and even necrosis (Ali et al., 2024). Accordingly, the MTX-induced liver injury model is commonly employed for mechanistic studies of drug-induced liver injury and hepatoprotective drug screening.

Li Y. et al. (2021) demonstrated that DIO exerted significant protective effects against MTX-induced liver injury. DIO improved the viability of LO2 cells, reduced serum ALT and AST levels, and ameliorated histopathological liver injury (Li Y. et al., 2021). Meanwhile, DIO decreased intracellular ROS levels, increased the levels of SOD, GSH and GSH-Px, and reduced MDA content, confirming its potent antioxidant activity (Li Y. et al., 2021). Mechanistically, DIO alleviated MTX-induced oxidative stress via regulating the miR-145–5p/Sirt5 signaling axis (Li Y. et al., 2021). DIO downregulated miR-145–5p expression, thereby relieving its inhibitory effect on Sirt5, subsequently upregulating the expression of antioxidant-related proteins including SOD1, Nrf2, GST, HO-1, GCLC, and NQO1, while modulating the activity of the Keap1/Nrf2 pathway (Li Y. et al., 2021). Furthermore, miR-145–5p mimic or Sirt5 silencing aggravated MTX-induced oxidative stress and liver injury, whereas DIO markedly reversed these alterations, suggesting that its hepatoprotective effects are closely associated with the regulation of miR-145-5p-mediated oxidative stress (Li Y. et al., 2021).

#### 3.2.2 Acetaminophen

Acetaminophen (APAP) is a widely used antipyretic and analgesic drug; however, overdose may cause severe liver injury (Hinz et al., 2024). APAP is metabolized by cytochrome P450 enzymes, particularly CYP2E1, to generate the highly reactive metabolite N-acetyl-p-benzoquinone imine (NAPQI) (Liao et al., 2023). When cellular GSH is depleted, NAPQI covalently binds to intracellular proteins, triggering oxidative stress, mitochondrial dysfunction, and hepatocyte apoptosis and necrosis, ultimately leading to acute liver injury (Liao et al., 2023). Consequently, APAP-induced liver injury models have been widely employed to investigate drug-induced hepatotoxicity.

Zhao et al. (2012) reported that DIO markedly improved AST release, mitochondrial dysfunction, apoptosis, and necrosis in APAP-treated HepG2 cells, and significantly attenuated histopathological liver injury, hepatocyte apoptosis, and mitochondrial edema in mice. Mechanistically, DIO inhibited APAP-induced CYP2E1 expression and activation, thereby reducing the production of toxic metabolites (Zhao et al., 2012). In addition, DIO modulated multiple parameters related to mitochondrial function, including reduction of mitochondrial Ca^2+^ levels, improvement of ATP2A2 expression, and attenuation of mitochondrial cardiolipin damage (Zhao et al., 2012). Moreover, DIO upregulated the expression of Bcl-2 and Bid, while downregulating Bax, Bak, and p53 expression, thereby alleviating hepatocyte apoptosis and necrosis (Zhao et al., 2012). Further proteomic analysis identified several potential target proteins, including Suox, Krt18, Rgn, Prdx1, MDH, and PNP, supporting that DIO exerts hepatoprotective effects primarily via the regulation of mitochondrial function and apoptosis (Zhao et al., 2012).

#### 3.2.3 Doxorubicin

Doxorubicin (Dox) is a classical anthracycline antitumor agent widely used in the treatment of various malignancies (Vitale et al., 2024). However, its clinical application is frequently accompanied by significant hepatotoxicity. Existing studies have confirmed that Dox induces excessive ROS overproduction, leading to oxidative stress, lipid peroxidation, and mitochondrial dysfunction. Concurrently, Dox activates signaling pathways associated with inflammation and apoptosis, ultimately resulting in hepatocyte injury and necrosis (Li H. et al., 2024; Li Y. et al., 2024). Accordingly, Dox-induced liver injury models are extensively applied in preclinical and mechanistic studies of drug-induced hepatotoxicity and candidate hepatoprotective agents.

Notably, DIO markedly ameliorated AML-12 cell injury, reduced ROS levels and apoptosis, and significantly decreased ALT, AST, and MDA levels *in vivo*, while increasing the levels of antioxidant indicators, including SOD, GSH, and GSH-Px, ultimately alleviating hepatic injury (Song et al., 2019). Mechanistic studies demonstrated that DIO promoted the nuclear translocation of Nrf2, upregulated the expression of Sirt1 and HO-1 while suppressing that of FOXO1 and Keap1, thereby attenuating oxidative stress (Song et al., 2019). Furthermore, DIO inhibited NF-κB nuclear translocation and reduced the expression of inflammatory cytokines, including TNF-α, IL-1β, and IL-6 (Song et al., 2019). Collectively, these findings suggest that DIO protects against Dox-induced liver injury primarily through modulation of the Sirt1/FOXO1/NF-κB signaling pathway (Song et al., 2019).

### 3.3 Immune-mediated liver injury

Immune-mediated liver injury is closely associated with excessive activation of innate immunity and massive release of inflammatory cytokines (Triantafyllou et al., 2025). Lipopolysaccharide (LPS) can activate the TLR4/MyD88/NF-κB signaling pathway, thereby inducing the excessive production of pro-inflammatory cytokines, including TNF-α, IL-1β, and IL-6, ultimately resulting in severe hepatic inflammatory injury (Kawaratani et al., 2013). In addition, during systemic inflammatory response syndrome (SIRS), TLR2/MyD88-mediated inflammatory cascades also contribute to liver damage and multiple organ dysfunction (van der Poll and Meijers, 2010). Thus, LPS- and zymosan-induced models are widely applied in preclinical research of immune-mediated inflammatory liver injury.

Yao et al. (2016) demonstrated that DIO markedly reduced serum ALT and AST levels, alleviated inflammatory liver injury and hepatocellular necrosis, and improved LPS-induced injury in AML-12 and HepG2 cells. Subsequent mechanistic investigations revealed that DIO significantly downregulated the expression of inflammation-associated signaling molecules, including TLR4, MyD88, IRAK1, TRAF6, p-IKK, p-IκBα and p-NF-κB p65, and concurrently decreased the levels of HMGB1, IL-1β, IL-6 and TNF-α, thereby suppressing TLR4/MyD88/NF-κB-mediated inflammatory responses (Yao et al., 2016).

Furthermore, in mouse models of zymosan-induced systemic inflammation, DIO also exhibited potent anti-inflammatory and organ-protective effects (Zhao et al., 2018). DIO attenuated inflammatory cell infiltration and tissue necrosis in the liver, kidney and intestine, reduced ALT, AST, MDA and MPO levels, increased SOD activity, and inhibited macrophage migration (Zhao et al., 2018). Mechanistically, DIO suppressed the TLR2/MyD88/NF-κB signaling pathway by downregulating the expression of TLR2, MyD88, NF-κB and HMGB1, and concurrently reduced the levels of IL-1β, IL-6 and TNF-α (Zhao et al., 2018). Collectively, these findings suggest that DIO ameliorates immune-mediated liver injury by modulating the TLR2/MyD88-dependent inflammatory signaling pathways (Zhao et al., 2018).

### 3.4 Metabolism-related liver injury

Metabolism-related liver injury is closely associated with lipid metabolism disorders, cholesterol accumulation, and abnormal bile acid metabolism (Chiang and Ferrell, 2018). Hyperuricemia not only promotes oxidative stress and inflammatory responses, but also aggravates dysregulation of cholesterol metabolism, thereby accelerating the progression of atherosclerosis and metabolism-related liver injury (Dasgupta et al., 2023). Hence, targeting the cholesterol-bile acid metabolic pathway is considered an important strategy for ameliorating metabolism-related liver injury.

Interestingly, DIO has been reported to significantly ameliorate hyperuricemia-associated lipid metabolism disorders and attenuate the progression of atherosclerosis (Bao et al., 2022). DIO reduced elevated serum cholesterol levels under hyperuricemic conditions, and these beneficial effects were closely associated with the regulation of the hepatic FXR/SHP/CYP7A1 signaling pathway (Bao et al., 2022). Further studies demonstrated that tigogenin, a primary bioactive metabolite of DIO, suppressed FXR activation and upregulated CYP7A1 expression, thereby promoting the conversion of cholesterol into bile acids and accelerating cholesterol metabolism (Bao et al., 2022). Moreover, clinical trials revealed that DIO-enriched preparations effectively reduced serum cholesterol levels in patients with hyperuricemia, suggesting the therapeutic potential of DIO in metabolism-related liver injury and lipid metabolic disorders (Bao et al., 2022). The hepatoprotective effects of DIO against liver injury and its underlying mechanisms are summarized in Table 2.

**TABLE 2:** Hepatoprotective effects and underlying mechanisms of DIO in liver injury.

Models	Types	Routes	Dosages of administration	Molecular mechanisms	Years	References
CCl_4_-induced liver injury in male Kunming mice	*In vivo*	i.g	100 mg/kg DIO for 5 days	Regulating the expression of HSPA5, ANXA6, IVD, RPS6, Cygb and NDPK-A	2010	Lu et al. (2011)
CCl_4_-induced acute liver injury in male Kunming mice	*In vivo*	i.g	100 mg/kg DIO for 5 days	Through inhibiting lipid peroxidation, inflammatory cytokines, necrosis and apoptosis,	2012	Lu et al. (2012)
CCl_4_-induced acute liver injury in male Kunming mice	*In vivo*	i.g	25, 50, 100 mg/kg DIO-NS for 7 days	N/A	2019	Ju et al. (2019)
TAA-induced acute liver injury in male Sprague-Dawley rats	*In vivo*	i.g	15, 30, 60 mg/kg DIO for 7 days	Through inhibiting oxidative stress and in ammation via FXR/AMPK signal pathway	2018	Zheng et al. (2018)
TAA-induced acute liver injury in mice	*In vivo*	i.g	20, 40, 80 mg/kg DIO for 7 days			
TAA-induced acute liver injury in male rats	*In vivo*	i.g	25, 50, 100 mg/kg DIO for 7 days	Activation of the FXR/AMPK signaling pathway	2021	Liu et al. (2021)
DMN-induced acute liver injury in male Sprague-Dawley rats	*In* *vivo*	i.g	15, 30, 60 mg/kg DIO for 7 days	Through regulating apoptosis, oxidative stress and inflammation	2016	Zhang et al. (2016c)
DMN-induced acute liver injury in male C57BL/6 mice	*In vivo*	i.g	20, 40, 80 mg/kg DIO for 7 days			
MTX-treated NRK-52 E cells	*In vitro*	N/A	25–400 nM DIO for 6–48 h	Through regulating miR-145-5p-medicated oxidative stress	2021	Li et al. (2021b)
MTX-treated LO2 cells	*In vitro*	N/A	25–400 nM DIO for 6–48 h			
MTX-induced liver and kidney damages in male Sprague-Dawley rats	*In vivo*	i.g	15, 30, 60 mg/kg DIO for 7 days			
APAP-treated HepG2 cells	*In vitro*	N/A	0.65, 1.3, 2.6 μg/mL DIO for 6 h	Regulation of mitochondrial function	2012	Zhao et al. (2012)
APAP-induced acute liver damage in male Kunming mice	*In vivo*	i.g	25, 50, 100 mg/kg DIO for 5 days			
Dox-treated AML-12 cells	*In vitro*	N/A	50, 100, 200 ng/mL DIO for 24 h	Regulation of Sirt1/FOXO1/NF-κb signal	2019	Song et al. (2019)
Dox-induced acute liver damage in male C57BL/6 J mice	*In vivo*	i.g	15, 30, 60 mg/kg DIO for 14 days			
LPS-treated AML-12 cells	*In vitro*	N/A	150, 300, 600 ng/mL DIO for 24 h	Regulating TLR4/MyD88 signal pathway	2016	Yao et al. (2016)
LPS-treated HepG2 cells	*In vitro*	N/A	200, 400, 800 ng/mL DIO for 24 h			
LPS-induced inflammatory liver injury in male C57BL/6 J mice	*In vivo*	i.g	20, 40, 80 mg/kg DIO for 7 days			
LPS-induced inflammatory liver injury in male Wistar rats	*In vivo*	i.g	15, 30, 60 mg/kg DIO for 7 days			
LPS/Pam3CSK4-stimulated THP-1 cells	*In vitro*	N/A	57.5, 115, 230 ng/mL DIO for 24 h	Regulation of TLR2/MyD88 signal pathway	2018	Zhao et al. (2018)
Zymosan-induced SIRS in male C57BL/6 J mice	*In vivo*	i.g	20, 40, 80 mg/kg DIO for 7 days			
Zymosan-induced SIRS in male Sprague-Dawley rats	*In vivo*	i.g	15, 30, 60 mg/kg DIO for 7 days			
Potassium oxonate-treated ApoE^−/−^ mice	*In vivo*	i.g	100 mg/kg DIO for 3 months	Through FXR-Signaling Pathway	2022	Bao et al. (2022)

## 4 The hepatoprotective effects and mechanisms of DIO in MASLD

Accumulating evidence has demonstrated that DIO exerts significant protective effects against MASLD. The underlying mechanisms involve the regulation of lipid metabolism, improvement of energy metabolic disorders, attenuation of oxidative stress and inflammatory responses, promotion of autophagy and mitophagy, as well as alleviation of mitochondrial damage and hepatocyte apoptosis.

Dysregulated lipid metabolism is a central event in the initiation and progression of MASLD, and DIO has been shown to markedly ameliorate hepatic lipid accumulation and lipid metabolic dysregulation. Yao et al. (2018) reported that DIO significantly reduced hepatic lipid deposition and improved serum and hepatic biochemical parameters in primary hepatocytes, AML-12 and HepG2 cells, as well as in high-fat diet (HFD)-induced mouse and rat models. Mechanistically, DIO primarily regulated the expression of lipid metabolism-related factors through activation of the SIRT1/AMPK signaling pathway, including downregulation of lipogenesis-associated proteins such as SREBP-1c, FAS, and SCD, along with upregulation of fatty acid oxidation-related factors including CPT, FoxO1, and ATGL (Yao et al., 2018). Collectively, these effects suppressed lipid synthesis while promoting fatty acid β-oxidation. Furthermore, the beneficial effects of DIO on lipid metabolism were markedly attenuated following treatment with the SIRT1 inhibitor nicotinamide or the AMPK inhibitor Compound C, which further confirmed the pivotal role of the SIRT1/AMPK signaling pathway in the anti-MASLD activity of DIO (Yao et al., 2018).

In addition to improving lipid metabolism, the regulatory effects of DIO on systemic energy homeostasis and hepatic oxidative injury have also attracted increasing attention. Liu et al. (2015a) demonstrated that in HFD-induced C57BL/6 J mice and ob/ob mice, DIO significantly reduced body weight gain and hepatic lipid accumulation, enhanced oxygen consumption and energy expenditure, and improved serum and hepatic biochemical parameters. Meanwhile, DIO markedly attenuated oxidative stress and inflammatory responses, inhibited triglyceride and cholesterol synthesis, and promoted fatty acid β-oxidation (Liu et al., 2015a). Further mechanistic studies revealed that DIO suppressed the phosphorylation of the MAPK signaling pathway and induced autophagy, thereby ameliorating hepatic steatosis (Liu et al., 2015a). These findings suggest that DIO not only regulates hepatic lipid metabolism but also delays MASLD progression by enhancing energy metabolism and reducing oxidative injury.

In recent years, mitochondrial dysfunction has emerged as a critical contributor to MASLD pathogenesis, and the protective role of DIO in maintaining mitochondrial homeostasis has been further elucidated. Given the close association between mitochondrial injury and fatty liver hemorrhagic syndrome, Xing et al. (2024) found that DIO markedly alleviated mitochondrial damage, hepatic lipid droplet accumulation, oxidative stress, and hepatocyte apoptosis in a high-energy and low-protein diet-induced model. Mechanistically, DIO promoted mitophagy and enhanced autophagic flux via activating the ERα/AMPK signaling pathway, thereby maintaining mitochondrial homeostasis and alleviating hepatocellular injury (Xing et al., 2024). Moreover, treatment with the lysosomal acidification inhibitor bafilomycin A1 or the ERα-specific inhibitor methylpiperidino pyrazole significantly weakened the protective effects of DIO on mitochondrial function and hepatocyte apoptosis, further confirming that ERα/AMPK-mediated mitophagy is indispensable for DIO to ameliorate fatty liver disease (Xing et al., 2024). The hepatoprotective effects and underlying mechanisms of DIO in MASLD are summarized in Table 3.

**TABLE 3:** Hepatoprotective effects and underlying mechanisms of DIO in MASLD.

Models	Types	Routes	Dosages of administration	Molecular mechanisms	Years	References
High-energy and low-protein diet-induced yline Brown laying hens	*In vivo*	p.o	100, 500, 1,000 mg/kg DIO for 12 weeks	Activation of the ERα-mediated AMPK-mTOR signaling pathway	2024	Xing et al. (2024)
Palmitic acid-stimulated LMH chicken cells	*In vitro*	N/A	0.3 nM DIO for 12 h			
Palmic acid-treated primary cultured hepatocytes	*In vitro*	N/A	125, 250, 500 ng/mL DIO for 24 h	Regulation of lipid metabolism via the SIRT1/AMPK signaling pathway	2018	Yao et al. (2018)
Palmic acid-treated AML-12 cells	*In vitro*	N/A	150, 300, 600 ng/mL DIO for 24 h			
Palmic acid-treated HepG-2 cells	*In vitro*	N/A	200, 400, 800 ng/mL DIO for 24 h			
HFD-induced Wistar male rats	*In vivo*	p.o	15, 30, 60 mg/kg DIO for 8 weeks			
HFD-induced C57BL/6 J male mice	*In vivo*	p.o	20, 40, 80 mg/kg DIO for 8 weeks			
HFD-induced C57BL/6 J mice	*In* *vivo*	i.g	20, 40, 80 mg/kg DIO for 10 weeks	Inhibition of fatty acid synthesis, promotion of fatty acid β-oxidation, attenuation of oxidant stress and inflammation, regulation of the MAPK signaling pathway and induction of autophagy	2015	Liu et al. (2015a)
ob/ob mice	*In vivo*	i.g	80 mg/kg DIO for 8 weeks			
Bel-7402 cells	*In vitro*	N/A	1, 2 μM DIO for 24 h			

## 5 The hepatoprotective effects and mechanisms of DIO in cholestatic liver diseases

Bile acid transport dysfunction is a critical event in the initiation and progression of intrahepatic cholestasis (Bjornsson and Devarbhavi, 2025; Fuchs et al., 2025). Zhang A. et al. (2016) demonstrated that in an α-naphthylisothiocyanate (ANIT)-induced cholestatic mouse model, DIO markedly reduced serum levels of ALT, AST and bilirubin, while alleviating histopathological liver injury. Further investigations revealed that DIO restored the impaired uptake function of organic anion transporting polypeptides (OATPs) under cholestatic conditions, prevented the adaptive downregulation of OATP1A1 and OATP1B2, and promoted the upregulation of OATP1A4, multidrug resistance-associated protein 2 (MRP2), and bile salt export pump (BSEP). These effects collectively facilitated bile acid efflux and reduced intrahepatic bile acid accumulation (Zhang A. et al., 2016). In addition, DIO activated FXR signaling and enhanced the expression of SHP, which further coordinately regulated MRP2 and BSEP expression to maintain bile acid transport homeostasis (Zhang A. et al., 2016).

In addition to regulating bile acid transport, DIO has been demonstrated to attenuate cholestasis-associated oxidative stress and hepatocyte apoptosis. Specifically, DIO significantly restored the levels of GSH, GSH-Px and SOD, while reducing MDA content and ROS generation (Yao et al., 2017). Moreover, DIO downregulated the expression of pro-apoptotic proteins, including Bak, Bax, Caspase-3 and Caspase-9, while upregulating the anti-apoptotic proteins Bcl-2 and Bcl-xL, thereby mitigating bile acid-induced hepatocyte apoptosis (Yao et al., 2017). Further mechanistic studies demonstrated that DIO suppressed the PI3K/Akt signaling pathway and enhanced the expression of antioxidant-related molecules, including Nrf2, GCLc, GCLm, NQO1 and HO-1, thereby strengthening cellular antioxidant defenses and alleviating liver injury (Yao et al., 2017). Collectively, these findings indicate that DIO ameliorates cholestatic liver disease through multiple coordinated mechanisms, including promotion of bile acid transport and excretion, attenuation of oxidative stress, and inhibition of hepatocyte apoptosis. These results highlight the potential of DIO as a promising natural therapeutic agent for the prevention and treatment of cholestatic liver injury. The hepatoprotective effects and underlying mechanisms of DIO in cholestatic liver diseases are summarized in Table 4.

**TABLE 4:** Hepatoprotective effects and underlying mechanisms of DIO in cholestatic liver diseases.

Models	Types	Routes	Dosages of administration	Molecular mechanisms	Years	References
ANIT-induced cholestatic hepatitis in male Wistar rats	*In vivo*	i.g	25, 50, 100 mg/kg DIO for 24 h	Upregulation of OATP, Mrp2 and Bsep expression and function	2016	Zhang et al. (2016a)
Sandwich-cultured hepatocytes	*In vitro*	N/A	200, 400, 800 ng/mL DIO for 24 h	Regulation of transporters, apoptosis and oxidative stress	2017	Yao et al. (2017)
ANIT-induced intrahepatic cholestasis in male Wistar rats	*In vivo*	i.g	15, 30, 60 mg/kg DIO for 7 days			
ANIT-induced intrahepatic cholestasis in male C57BL/6 J mice	*In vivo*	i.g	20, 40, 80 mg/kg DIO for 7 days			
Bel-7402 cells	*In vitro*	N/A	1, 2 μM DIO for 24 h			

## 6 The hepatoprotective effects and mechanisms of DIO in liver fibrosis

### 6.1 Wnt/β-catenin

The Wnt/β-catenin signaling pathway plays a pivotal role in HSC activation and liver fibrosis progression (Duspara et al., 2021). Interestingly, Zhang et al. (2015a) demonstrated that DIO markedly inhibited the proliferation and activation of HSC-T6 cells, LX-2 cells and primary rat HSCs, while exhibiting no obvious cytotoxicity against normal hepatocytes. Mechanistically, DIO upregulated PPAR-γ expression and downregulated the levels of fibrosis-related factors, including α-smooth muscle actin (α-SMA), TGF-β1, collagen type I alpha 1 (Col1a1) and Col3a1. This regulatory effect ultimately suppressed HSC activation and collagen deposition (Zhang et al., 2015a). In addition, DIO induced apoptosis and senescence in activated HSCs and promoted extracellular matrix (ECM) degradation, further alleviating liver fibrosis (Zhang et al., 2015a).

*In vivo* studies further demonstrated that DIO significantly ameliorated histopathological liver injury in CCl_4_-induced fibrotic rats, reduced fibrosis-related indicators such as hydroxyproline (HYP), laminin and α-SMA, and improved liver function parameters including AST and ALT (Zhang et al., 2015a). Meanwhile, DIO also attenuated oxidative stress and inflammatory responses, indicating its potent hepatoprotective effects (Zhang et al., 2015a).

Further investigations revealed that the Wnt/β-catenin signaling pathway is a core mechanism underlying the anti-fibrotic effects of DIO (Song and Yu, 2022). DIO markedly downregulated the expression of Wnt1, β-catenin, and their downstream target genes, including c-Myc and Cyclin D1, thereby suppressing the activation of the Wnt/β-catenin signaling pathway (Song and Yu, 2022). This suppression ultimately reduced HSC proliferation and activation, leading to decreased ECM deposition. Additionally, DIO exerted its anti-hepatic fibrosis effects by coordinately regulating the TGF-β1/Smad, MAPK, and mitochondria-associated signaling pathways (Zhang et al., 2015a).

### 6.2 PI3K/Akt

The PI3K/Akt signaling pathway serves a critical function in the pathogenesis of liver fibrosis (Shamsan et al., 2024). Activation of this pathway promotes HSC proliferation and activation, enhances collagen synthesis and ECM deposition, and aggravates inflammatory responses (Schwabe and Brenner, 2025). In addition, PI3K/Akt signaling interacts with multiple profibrotic pathways, including TGF-β1/Smad signaling, thereby accelerating hepatic fibrogenesis (Parola and Pinzani, 2019). Therefore, inhibition of PI3K/Akt signaling is considered an important therapeutic strategy for liver fibrosis.

Xu et al. (2017) demonstrated that DIO exerts significant anti-fibrotic effects by regulating the PI3K/Akt signaling pathway. Stable-isotope labeling by amino acids in cell culture-based proteomic analysis identified 121 differentially expressed proteins in DIO-treated LX-2 cells (Xu et al., 2017). Mechanistically, DIO significantly downregulated fibronectin, focal adhesion kinase 1, ITGA5, p-PI3K/PI3K, p-Akt/Akt and p-mTOR/mTOR expression, and downregulated collagen-related genes, including Col1a1, Col1a2, Col2a1, Col5a1 and Col6a1 (Xu et al., 2017). Molecular docking and transfection experiments further confirmed that ITGA5 is a functional target of DIO, mediating its inhibitory effects on PI3K/Akt signaling and collagen synthesis (Xu et al., 2017).

Consistent with the *in vitro* observations, in a CCl_4_-induced mouse model of liver fibrosis, DIO significantly ameliorated hepatic histopathological injury and reduced serum glutamate pyruvate transaminase, HYP, TNF-α and liver index levels (Lu et al., 2021). Moreover, DIO markedly decreased the expression of TGF-β1 and p-Akt/Akt, indicating effective suppression of PI3K/Akt signaling (Lu et al., 2021). Collectively, these findings indicate that DIO suppresses HSC activation, collagen deposition, and inflammatory responses by inhibiting the PI3K/Akt signaling pathway, which underscores its potential as a promising natural therapeutic candidate for liver fibrosis.

### 6.3 Sirt1/Nrf2

Oxidative stress plays a critical role in HSC activation and ECM accumulation during liver fibrosis (Bellanti et al., 2023). The Sirt1/Nrf2 signaling pathway serves as a key antioxidant regulatory axis that protects against hepatic fibrogenesis by upregulating antioxidant enzymes, reducing oxidative stress, and suppressing HSC activation. Accordingly, this pathway represents a promising therapeutic target for liver fibrosis (Li W. et al., 2024; Mao et al., 2024).

Previous studies demonstrated that DIO exerts anti-fibrotic effects through modulation of the Sirt1/Nrf2 signaling pathway (Gu et al., 2016). In bile duct ligation (BDL)- and DMN-induced liver fibrosis models, DIO markedly alleviated hepatic fibrosis and inhibited HSC activation (Gu et al., 2016). Mechanistically, DIO promoted Nrf2 nuclear translocation and upregulated the expression of Sirt1, HO-1, GST, GCLC and GCLM, while suppressing p38 MAPK phosphorylation and reducing the levels of fibrosis-related markers, including Col1a1, Col3a1, α-SMA and fibronectin (Gu et al., 2016). Furthermore, siRNA-mediated silencing of Sirt1 and Nrf2, combined with intervention using the p38 MAPK inhibitor SB-203580, further confirmed the involvement of the Sirt1/Nrf2/p38 MAPK signaling pathway in the anti-fibrotic effects of DIO (Gu et al., 2016). Collectively, these findings suggest that DIO attenuates oxidative stress and HSC activation through regulation of the Sirt1/Nrf2 pathway, thereby exerting potent anti-hepatic fibrotic effects.

### 6.4 TLR4/MyD88/NF-κB

The TLR4/MyD88/NF-κB signaling pathway plays a crucial role in the initiation and progression of liver fibrosis (Hammerich and Tacke, 2023). During hepatic injury, activation of TLR4 recruits MyD88, which subsequently activates the NF-κB signaling pathway and promotes the release of multiple pro-inflammatory cytokines, including IL-1, IL-6 and TNF-α (Hammerich and Tacke, 2023). These events further induce HSC activation, collagen deposition and excessive ECM accumulation (Guo and Friedman, 2010). In addition, the TLR4/MyD88/NF-κB pathway interacts with classical pro-fibrotic signaling pathways such as TGF-β1, thereby accelerating hepatic fibrogenesis (Guo et al., 2024). Accordingly, targeting the TLR4/MyD88/NF-κB signaling pathway is considered an important therapeutic strategy for liver fibrosis.

Liu et al. (2015b) demonstrated that DIO markedly ameliorated alcoholic liver fibrosis by regulating the TLR4/MyD88/NF-κB signaling pathway. In mouse models of alcohol-induced liver fibrosis and LPS-stimulated HSC-T6 and LX-2 cells, DIO significantly inhibited HSC activation, reduced collagen deposition, and attenuated inflammatory responses (Liu et al., 2015b). Mechanistically, DIO downregulated the expression of TLR4, MyD88 and NF-κB, while decreasing the levels of inflammatory and fibrosis-related factors, including IL-1, IL-6, TNF-α, TGF-β1, α-SMA and Col1a1 (Liu et al., 2015b). Furthermore, TLR4 overexpression partially reversed the anti-fibrotic effects of DIO, whereas treatment with the MyD88 inhibitor ST2825 or the NF-κB inhibitor pyrrolidine dithiocarbamate markedly attenuated the inhibitory effects of DIO on TGF-β1, α-SMA and Col1a1 expression (Liu et al., 2015b). These findings further confirm that the TLR4/MyD88/NF-κB signaling pathway is critically required for the anti-fibrotic activity of DIO.

### 6.5 SDC-4

Syndecan-4 (SDC-4) is a critical cell adhesion molecule involved in cell migration, adhesion and ECM remodeling (Sharip and Kunz, 2025). Emerging evidence indicates that SDC-4 promotes HSC migration and activation through regulation of signaling molecules such as fibronectin, PKCα, Src, FAK and ERK1/2, thereby accelerating the progression of liver fibrosis (Longmate et al., 2024; Zhang et al., 2024c). Therefore, targeting SDC-4-mediated migration-related signaling pathways may serve as a promising therapeutic strategy for liver fibrosis.

Yin et al. (2017) demonstrated that DIO significantly inhibited HSC migration through modulation of the SDC-4-dependent signaling pathway, thereby exerting anti-fibrotic effects. Quantitative proteomic analysis based on isobaric tags for relative and absolute quantitation (iTRAQ) identified 1,566 differentially expressed proteins in DIO-treated HSC-T6 cells (Yin et al., 2017). Further mechanistic studies revealed that DIO markedly suppressed HSC-T6 cell migration and adhesion, while concurrently downregulating the expression of migration-related proteins, including fibronectin, PKCα, Src, FAK and ERK1/2 (Yin et al., 2017). Moreover, shRNA-mediated knockdown of SDC-4 significantly reduced HSC-T6 cell migration, an inhibitory effect that was further enhanced by DIO treatment (Yin et al., 2017). Collectively, these findings suggest that DIO suppresses HSC migration by targeting SDC-4-related signaling pathways, thereby alleviating liver fibrosis. They also indicate that SDC-4 may serve as an important therapeutic target mediating the anti-fibrotic effects of DIO.

### 6.6 Others

In addition to regulating classical signaling pathways such as Wnt/β-catenin, PI3K/Akt, Sirt1/Nrf2, TLR4/MyD88/NF-κB and SDC-4, DIO also exerts anti-hepatic fibrotic effects through multiple targets and biological processes. A proteomic analysis based on two-dimensional differential in-gel electrophoresis revealed that DIO modulated several novel fibrosis-related biomarkers in CCl_4_-induced liver fibrosis models, including PDIA3, SBP1, GS, SMP30, hemopexin, keratin 8, keratin 18, vimentin, Annexin A5, and dermatopontin (Zhang et al., 2015b). These proteins are mainly involved in oxidative stress, cellular senescence, cytoskeletal remodeling, inflammatory responses and ECM regulation, suggesting that DIO may coordinately regulate liver fibrosis through multiple pathways and targets. The hepatoprotective effects and underlying mechanisms of DIO in liver fibrosis are summarized in Table 5.

**TABLE 5:** Hepatoprotective effects and underlying mechanisms of DIO in liver fibrosis.

Models	Types	Routes	Dosages of administration	Molecular mechanisms	Years	References
HSC-T6 cells	*In vitro*	N/A	1.25, 2.5, 5 ng/mL DIO for 12–48 h	Modulation of the TGF-β1/Smad Wnt/β-catenin, MAPK and mitochondrial signaling pathways	2015	Zhang et al. (2015a)
LX-2 cells	*In vitro*	N/A	1.25, 2.5, 5 ng/mL DIO for 12–48 h			
Primary HSCs cells	*In vitro*	N/A	1.25, 2.5, 5 ng/mL DIO for 12–48 h			
CCl_4_-induced liver fibrosis in male Wistar rats	*In vivo*	i.g	20, 40, 60 mg/kg DIO for 7 weeks			
CCl_4_-induced liver fibrosis in male Wistar rats	*In vivo*	i.g	20, 40, 60 mg/kg DIO for 6 weeks			
CCl_4_-induced liver fibrosis in male Sprague-Dawley rats	*In vivo*	i.g	50 mg/kg DIO for 5 days	Inhibition of the Wnt/β-catenin signaling pathway	2022	Song and Yu (2022)
LX-2 cells	*In vitro*	N/A	0.6, 1.2, 2.4 μg/mL DIO for 24 h	Modulation of the PI3K/Akt signaling pathway	2017	Xu et al. (2017)
DMN-induced liver fibrosis in rats	*In vivo*	i.g	20, 40, 60 mg/kg DIO for 6 weeks			
CCl_4_-induced liver fibrosis in C57BL/6 J mice	*In vivo*	i.g	50, 100 mg/kg DIO for 3 weeks	Blockade of the PI3K/Akt signaling pathway	2021	Lu et al. (2021)
HSC-T6 cells	*In vitro*	N/A	0.6, 1.2, 2.4 μg/mL DIO for 24 h	Inhibition of the p38 MAPK pathway via Sirt1/Nrf2 signaling	2016	Gu et al. (2016)
LX-2 cells	*In vitro*	N/A	0.6, 1.2, 2.4 μg/mL DIO for 24 h			
BDL-induced liver fibrosis in male Sprague-Dawley rats	*In vivo*	i.g	20, 40, 60 mg/kg DIO for 4 weeks			
DMN-induced liver fibrosis in male Sprague-Dawley rats	*In vivo*	i.g	20, 40, 60 mg/kg DIO for 6 weeks			
HSC-T6 cells	*In vitro*	N/A	0.25, 0.5, 1 μg/mL DIO for 24 h	Regulation of the TLR4/MyD88/NF-κB signaling pathway	2015	Liu et al. (2015b)
LX-2 cells	*In vitro*	N/A	0.125, 0.25, 0.5 μg/mL DIO for 24 h			
Alcoholic-induced liver fibrosis in male C57BL/6 J mice	*In vivo*	i.g	20, 40, 80 mg/kg DIO for 14 weeks			
HSC-T6 cells	*In vitro*	N/A	1.25, 2.5, 5 μg/mL DIO for 24 h	Regulation of the SDC-4 signaling pathway	2017	Yin et al. (2017)
CCl_4_-induced liver fibrosis in male Wistar rats	*In vivo*	i.g	20, 40, 60 mg/kg DIO for 4 weeks	Modulation of multiple biological processes, including cytoskeleton, senescence, apoptosis, oxidative stress and inflammation	2015	Zhang et al. (2015b)

## 7 The hepatoprotective effects and mechanisms of DIO in liver cancer

Accumulating evidence has demonstrated that DIO exerts significant anti-tumor activity against HCC. The underlying mechanisms involve inhibition of tumor cell proliferation, induction of apoptosis, autophagy and ferroptosis, suppression of epithelial-mesenchymal transition (EMT), as well as reversal of multidrug resistance (MDR).

Apoptosis induction is a major mechanism underlying the anti-HCC effects of DIO. Zhang G. et al. (2016) reported that DIO inhibited the proliferation of Bel-7402 and HepG2 cells in a dose-dependent manner and induced typical apoptotic morphological alterations accompanied by DNA damage. Mechanistically, DIO upregulated the expression of pro-apoptotic proteins, including TP53, Bax and Caspase-3, while downregulating the anti-apoptotic protein Bcl-2, thereby activating the mitochondrial apoptosis pathway (Zhang G. et al., 2016). In addition, JC-1 staining demonstrated that DIO significantly reduced mitochondrial membrane potential, further promoting apoptosis (Liang et al., 2021). Notably, although DIO exhibited potent anti-proliferative effects against HCC cells, it also showed cytotoxicity toward normal hepatocytes (LO2 cells; IC_50_ = 2.04 μM), indicating that its potential hepatotoxicity requires careful evaluation in future therapeutic development (Liang et al., 2021).

In addition to inducing apoptosis, DIO also regulates tumor metabolic homeostasis and stress-response pathways. Among these, TP53-induced glycolysis and apoptosis regulator (TIGAR) has been identified as a critical molecular target (Mao et al., 2019). iTRAQ-based proteomic analysis revealed that TIGAR expression was markedly downregulated following DIO treatment in HCC cells (Mao et al., 2019). Further mechanistic studies demonstrated that DIO induced apoptosis, autophagy and DNA damage through modulation of TIGAR-associated signaling pathways (Mao et al., 2019). Specifically, DIO treatment increased the expression levels of p53, cleaved PARP, cleaved Caspase-3/9, Beclin-1 and LC3, while suppressing the activation of p-Akt, p-mTOR, CDK5, and p-ATM (Mao et al., 2019). These findings indicate that DIO interferes with glucose metabolism, autophagic activity, and DNA damage repair in HCC cells. Moreover, TIGAR silencing further enhanced the anti-tumor effects of DIO, confirming the critical role of the TIGAR-mediated p53, Akt/mTOR, and CDK5/ATM signaling pathways in its anti-HCC activity (Mao et al., 2019).

Besides apoptosis and autophagy, recent studies have demonstrated that DIO can induce ferroptosis in HCC cells. Teng et al. (2026) showed that DIO treatment significantly increased intracellular Fe^2+^, ROS and MDA levels in HepG2 cells, and induced changes consistent with the classical morphological features of ferroptosis: mitochondrial shrinkage and reduction of mitochondrial cristae. Mechanistically, DIO markedly downregulated the expression of SLC7A11 and GPX4 while upregulating transferrin receptor 1 (TFR1), suggesting that it induces ferroptosis by suppressing the SLC7A11/GPX4 antioxidant system, promoting lipid peroxidation and enhancing iron accumulation (Teng et al., 2026). Furthermore, treatment with the ferroptosis inhibitor Liproxstatin-1 partially reversed DIO-induced ROS accumulation and lipid peroxidation, further confirming that ferroptosis contributes to the anti-HCC effects of DIO (Teng et al., 2026).

Apart from promoting tumor cell death, DIO also suppresses the invasive and migratory capacities of HCC cells by inhibiting EMT progression. Chen et al. (2019) demonstrated that DIO significantly attenuated TGF-β1-induced invasion and migration in HepG2 cells. Correspondingly, DIO treatment upregulated the expression of epithelial markers such as E-cadherin, while markedly downregulating the expression of mesenchymal markers including N-cadherin, Vimentin, Snail and Slug (Chen et al., 2019). Further investigation revealed that DIO inhibited TGF-β1-induced phosphorylation of JNK, p38, and Erk (Chen et al., 2019). Importantly, the inhibitory effect of DIO on EMT was partially reversed by the p38 activator asiatic acid, indicating that suppression of the p38-MAPK pathway is a major mechanism by which DIO inhibits HCC cell invasion and metastasis (Chen et al., 2019).

Importantly, DIO has also shown promising potential in reversing multidrug resistance in HCC. Sun et al. (2011) reported that DIO significantly inhibited MDR1 promoter activity and reduced the drug-resistant phenotype of HepG2/adriamycin cells. Furthermore, DIO suppressed the expression of P-glycoprotein (P-gp) and enhanced intracellular accumulation of adriamycin in resistant HCC cells, thereby improving chemosensitivity (Sun et al., 2011). These findings suggest that DIO not only exerts direct anti-tumor effects, but may also serve as a potential chemosensitizer for overcoming MDR in HCC treatment. The hepatoprotective effects and underlying mechanisms of DIO in liver cancer are summarized in Table 6.

**TABLE 6:** Hepatoprotective effects and underlying mechanisms of DIO in liver cancer.

Models	Types	Routes	Dosages of administration	Molecular mechanisms	Years	References
HepG2/adriamycin cells	*In* *vitro*	N/A	2 μg/mL DIO for 24 h	Inhibition of the MDR1 promoter activity	2011	Sun et al. (2011)
Bel-7402 cells	*In vitro*	N/A	1–10 μM DIO for 24–48 h	Induction of apoptosis via the TP53/BAX/Caspase-3-mediated mitochondria pathway	2016	Zhang et al. (2016b)
HepG2 cells	*In vitro*	N/A	1–10 μM DIO for 24–48 h			
Lovo cells	*In vitro*	N/A	1–10 μM DIO for 24–48 h			
EAhy926 cells	*In vitro*	N/A	1–10 μM DIO for 24–48 h			
Male nude mice	*In vivo*	i.p	6, 12, 24 mg/kg DIO for 4 weeks			
SMMC7721 cells	*In vitro*	N/A	1.4, 2.9, 5.8 μM DIO for 24 h	Regulation of the TIGAR-mediated CDK5/ATM signaling pathway	2018	Mao et al. (2019)
HepG2 cells	*In vitro*	N/A	1.4, 2.9, 5.8 μM DIO for 24 h			
Male Wistar rats	*In vivo*	i.g	15, 30, 60 mg/kg DIO for 18 weeks			
BALB/c nude mice	*In vivo*	i.g	20, 40, 80 mg/kg DIO for 27 days			
HepG2 cells	*In* *vitro*	N/A	0.5, 1, 2 μM DIO for 24 h	Inhibition of the p38-MAPK signaling pathway	2019	Chen et al. (2019)
HepG2 cells	*In vitro*	N/A	2.5 μM DIO for 48 h	Inhibition of the SLC7A11/GPX4 signaling pathway	2026	Teng et al. (2026)
LO2 cells	*In vitro*	N/A	1, 2 μM DIO for 24 h	Upregulation of Bax expression, downregulation of Bcl-2 expression and induction of apoptosis	2021	Liang et al. (2021)
Bel-7402 cells	*In vitro*	N/A	1, 2 μM DIO for 24 h			

## 8 Toxicity of DIO

Current evidence indicates that DIO exhibits a relatively favorable safety profile under conventional oral administration. However, its toxicity demonstrates marked dose-, time-, sex- and species-dependent differences. Following long-term or high-dose exposure, the major toxicities are primarily associated with the liver, gastrointestinal tract and hematological system, while no significant pathological lesions have been consistently observed in vital organs including the heart, kidneys, and brain (Xu et al., 2012; Luo et al., 2018). Subchronic toxicity studies showed that male SD rats receiving 90-day oral DIO exposure experienced reduced food intake, decreased body weight gain, and mild gastrointestinal dilation, while female rats showed substantially higher tolerance to DIO exposure (Xu et al., 2012). Currently, the established toxicological mechanisms of DIO are considered to involve oxidative stress, mitochondrial dysfunction, bile acid metabolic disorders, apoptosis, and dysregulation of drug-metabolizing enzymes (Luo et al., 2018; Fang et al., 2022). In addition, DIO possesses hemolytic activity and inhibitory effects on cytochrome P450 enzymes, suggesting a potential risk of drug-drug interactions. Therefore, systematic elucidation of the toxicological characteristics of DIO is essential for its safe clinical application and subsequent pharmaceutical development.

### 8.1 Gastrointestinal toxicity

The gastrointestinal tract is one of the major target organs affected by long-term or high-dose DIO exposure. Animal studies demonstrated that male SD rats orally administered DIO at 300 mg/kg/d for 90 days developed mild gastrointestinal dilation, impaired gastrointestinal motility, and reduced digestive function, accompanied by decreased food intake and slower body weight gain (Xu et al., 2012). Clinically, gastrointestinal adverse reactions such as nausea, vomiting, and diarrhea have been occasionally reported in patients receiving DIO-containing preparations, namely, Di’ao Xinxuekang and DIO tablets (Xu et al., 2012; Fang et al., 2022). Further investigations revealed that DIO exerted potential cytotoxic effects on normal colonic epithelial cells by activating Caspase-3 and increasing the proportion of apoptotic cells in the Sub-G1 phase (Yum et al., 2010). These observations suggest that DIO-induced gastrointestinal toxicity may be associated with epithelial cell injury and impaired gastrointestinal motility.

### 8.2 Hepatotoxicity

Hepatotoxicity is currently recognized as the most critical safety concern associated with DIO and exhibits clear dose- and time-dependent characteristics. *In vitro* studies have demonstrated that DIO exerts dose-dependent toxicity toward normal human hepatocytes (LO2 cells) and HepG2 cells, with an IC_50_ value of approximately 2.25 μg/mL in LO2 cells. At a concentration of 10 μg/mL, DIO significantly elevated AST, ALP and LDH levels and induced hepatocyte apoptosis (Zhou et al., 2013; Zhang Y. et al., 2015). Mechanistic studies further indicated that DIO induces liver injury through multiple pathways, including increased LDH and MDA levels, decreased activities of Na^+^-K^+^-ATPase, GSH-PX and SOD, thereby triggering oxidative stress imbalance. Meanwhile, DIO suppressed the expression of sodium taurocholate co-transporting polypeptide and BSEP, leading to bile acid metabolic disorders (Yang et al., 2016). Moreover, DIO time-dependently upregulated CYP2E1 and CYP3A4 protein expression and induced mitochondrial structural and functional damage, further aggravating hepatocellular injury (Yang et al., 2016). A 2022 study based on a 3D HepG2 model further confirmed that DIO significantly suppressed CYP3A4 mRNA expression, thereby interfering with hepatic drug metabolism and increasing the risk of liver injury (Zhang et al., 2022).

*In vivo* studies have likewise confirmed the hepatotoxic potential of DIO. Elevated serum liver function markers and histopathological liver injury were observed in mice intravenously injected with 10 mg/kg/d DIO for 7 days, as well as in rats orally administered 300 mg/kg/d DIO for 90 days (Xu et al., 2012; Zhou et al., 2013). Moreover, occasional cases of elevated liver enzymes and drug-induced liver injury have been reported during clinical administration of DIO-containing preparations, although most patients recovered following drug withdrawal and hepatoprotective treatment (Luo et al., 2018; Fang et al., 2022). Taken together, current evidence indicates that DIO-induced hepatotoxicity involves multiple mechanisms, including oxidative stress, bile acid metabolic dysregulation, CYP enzyme imbalance, mitochondrial dysfunction, and apoptosis (Zhang Y. et al., 2015; Luo et al., 2018; Fang et al., 2022).

### 8.3 Metabolic interaction toxicity

In addition to direct hepatotoxicity, the interference of DIO with drug-metabolizing enzyme systems has also attracted considerable attention. Studies have demonstrated that DIO significantly inhibits the activities of key human hepatic cytochrome P450 enzymes, including CYP2C9, CYP2E1 and CYP3A4, with IC_50_ values of 22.60, 17.40 and 12.59 μM, respectively, primarily via a competitive inhibition mechanism (Tao et al., 2014). Notably, DIO also exhibits time-dependent inhibitory effects on CYP3A4 and further downregulates the expression of related CYP proteins (Tao et al., 2014). Given that CYP3A4 and CYP2C9 mediate the metabolism of a majority of clinically used drugs, DIO may increase the risk of drug accumulation and toxicity during combination therapy, particularly when co-administered with hepatotoxic agents or drugs with a narrow therapeutic window.

### 8.4 Hematological toxicity

Hemolysis is a well-documented adverse effect associated with saponin compounds. Long-term high-dose DIO exposure has been shown to induce mild hemolytic anemia, mainly characterized by reductions in erythrocyte count and hematocrit (Xu et al., 2012). As a steroidal saponin, DIO exhibits potent hemolytic activity, with a half-hemolytic concentration (HC_50_) of approximately 3.0–3.4 μM (Lin and Wang, 2010). Mechanistically, DIO can penetrate lipid bilayers and accumulate within lipid raft microdomains, where it interacts with membrane cholesterol, disrupts lipid raft homeostasis, alters membrane structure and fluidity, and ultimately induces erythrocyte membrane rupture and hemolysis (Lin and Wang, 2010).

**FIGURE 2:**
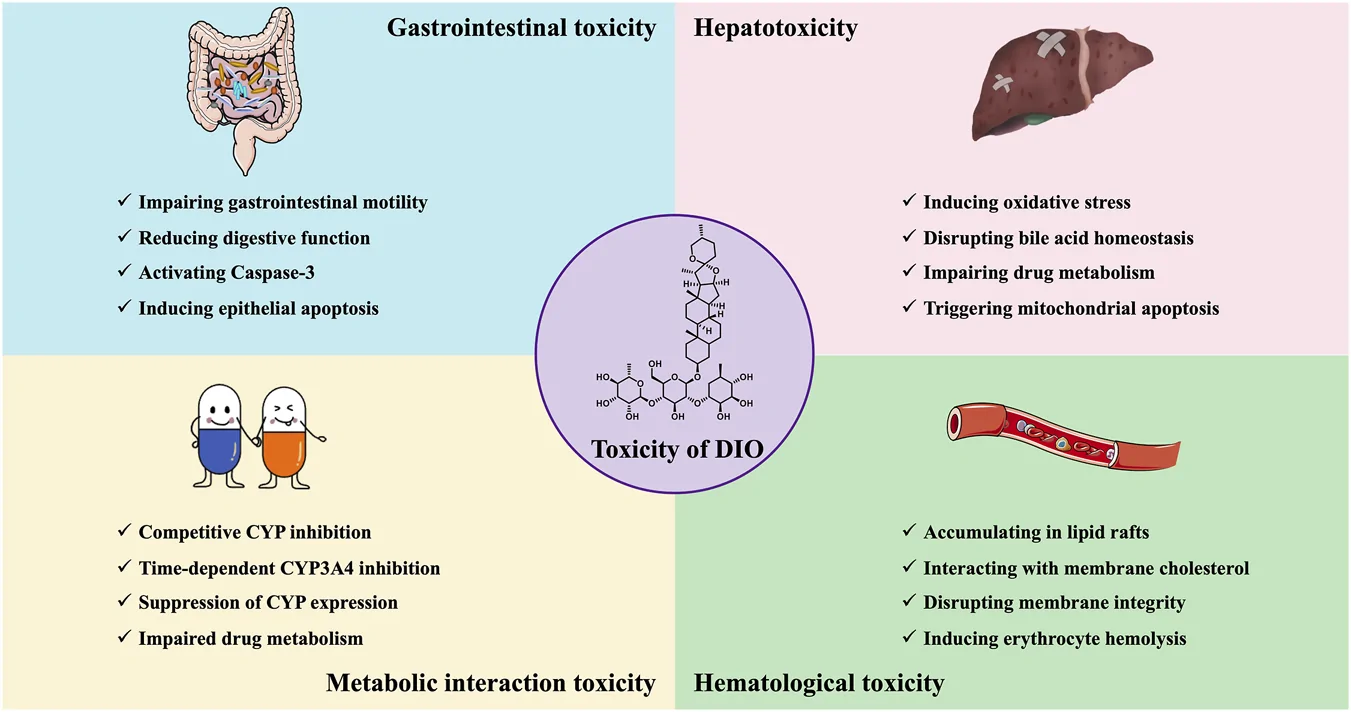
Schematic diagram of toxicity of DIO.

In recent years, nanodelivery strategies have been considered effective approaches for reducing the hemolytic toxicity of DIO. One study demonstrated that free DIO caused a hemolysis rate of up to 18.5% at 2.5 μg/mL, with an HC_50_ value of only 5.63 μg/mL. However, after DIO was formulated into cholesterol nanofibers and PEGylated nanocarriers, its HC_50_ values increased to 56.27 μg/mL and 116.50 μg/mL, respectively, indicating a marked reduction in hemolytic toxicity (Wang et al., 2022). Furthermore, *in vivo* studies showed that these nanocarriers effectively prevented hemolysis-associated organ damage induced by intravenous administration of free DIO while retaining its antitumor activity (Wang et al., 2022). These findings suggest that formulation optimization based on nanodelivery may represent a promising strategy for improving the clinical safety of DIO. A schematic illustration of DIO-induced toxicity is shown in Figure 2.

## 9 Pharmacokinetics of DIO

### 9.1 Absorption

Current evidence indicates that DIO exhibits slow absorption, extremely low oral bioavailability, and prolonged gastrointestinal retention following oral administration. Its absorption is jointly influenced by physicochemical properties, intestinal microbial metabolism and transporter-mediated efflux. Tang et al. (2015) analyzed metabolites in biological samples from rats orally administered DIO using UPLC-QTOF-MS and found that plasma concentrations increased gradually over time, while both the parent compound and its metabolites remained detectable for more than 36 h. These findings indicate the delayed absorption and prolonged intestinal retention of DIO.

Li et al. (2005) further compared the pharmacokinetic characteristics of DIO following intravenous and oral administration in rats. After intravenous injection (0.064–1.0 mg/kg), DIO exhibited a two-compartment pharmacokinetic model (Li et al., 2005). Increasing doses led to a marked reduction in systemic clearance, whereas the elimination half-life (t_1/2_β) and steady-state volume of distribution increased in a dose-dependent manner, without obvious enterohepatic circulation (Li et al., 2005). In contrast, oral administration (45 and 90 mg/kg) followed a one-compartment model, with an extremely low absolute bioavailability of only 0.21%–0.23% and a prolonged time to peak concentration (T_max_) of 16.1–19.4 h, indicating slow and sustained gastrointestinal absorption (Li et al., 2005).

The extremely low oral bioavailability of DIO results primarily from several factors. First, gut microbiota-derived glycosidases rapidly hydrolyze DIO into deglycosylated metabolites such as diosgenin, thereby markedly reducing the fraction of parent compound reaching systemic circulation (Lu et al., 2020; Peng et al., 2020). Second, the large molecular weight, poor lipophilicity, and low partition coefficient of DIO limit its transmembrane transport across intestinal epithelial lipid bilayers (Yu et al., 2012; Wang et al., 2022). Moreover, efflux transporters such as P-gp actively transport absorbed DIO back into the intestinal lumen, further restricting intestinal absorption (Liu et al., 2013; Manda et al., 2013). Therefore, improving the oral absorption and bioavailability of DIO has become an important focus in formulation optimization.

### 9.2 Distribution

Following systemic absorption, DIO exhibits extensive tissue distribution with marked organ selectivity—highest concentrations are observed in the liver and lungs, followed by the kidneys and spleen, whereas markedly lower levels are detected in the heart and brain (Li et al., 2005). At 3 h post-intravenous administration (1 mg/kg), hepatic DIO concentrations exceeded those in all other tissues, although the highest levels were detected in intestinal contents (Li et al., 2005). Hepatic concentrations declined slowly over 24–120 h, suggesting relatively slow tissue elimination (Li et al., 2005). After oral administration (90 mg/kg), peak concentrations in the liver, kidneys and lungs were reached at 24 h, with hepatic levels approximately 10-fold higher than plasma concentrations (Li et al., 2005). Moreover, detectable levels of DIO remained in intestinal contents even after 120 h, further indicating prolonged gastrointestinal retention (Li et al., 2005).

Lu et al. (2020) reported that several steroidal saponins, including DIO, were primarily distributed in the liver and lungs following either oral or intravenous administration, whereas levels in other tissues remained relatively low. Although some compounds could cross the blood-brain barrier, their concentrations in brain tissue were far below therapeutic levels (Lu et al., 2020).

### 9.3 Metabolism

DIO primarily undergoes phase I and phase II metabolic reactions *in vivo*, with deglycosylation, oxidation, and glucuronidation representing the major metabolic pathways (Tang et al., 2015; Zhu et al., 2015). Deglycosylation is the initial and rate-limiting step and is catalyzed primarily by intestinal microbiota and hepatic glycosidases (Li et al., 2005; Zhu et al., 2015). Through sequential hydrolysis of rhamnose and glucose residues, DIO is converted into metabolites such as diosgenin, thereby significantly influencing its biological activity and subsequent elimination (Li et al., 2005; Zhu et al., 2015).

Subsequently, DIO undergoes oxidative metabolism mediated by hepatic CYP450 enzymes, with CYP3A4 identified as the predominant isoform responsible for the formation of multiple hydroxylated metabolites (Tao et al., 2014). These metabolites are further conjugated via UDP-glucuronosyltransferases, yielding more water-soluble glucuronide conjugates that are readily excreted (Zhu et al., 2015). UPLC-QTOF-MS-based metabolomic analysis identified eight major metabolites, including seven phase I metabolites and one phase II glucuronide conjugate, highlighting glucuronidation as a key detoxification and elimination pathway for DIO (Zhu et al., 2015).

In addition, DIO acts as an inhibitor of CYP3A4 and CYP2C9, thereby posing a clinically significant risk for pharmacokinetic drug-drug interactions (Tao et al., 2014). Besides metabolic enzymes, transporters also participate in its disposition. OATPs have been identified as important transporters mediating hepatic uptake and accumulation of DIO, which may contribute to its preferential hepatic distribution (Zhang et al., 2013).

### 9.4 Excretion

Excretion studies demonstrated that feces represent the primary elimination route for unchanged DIO following both intravenous and oral administration, whereas only trace amounts of the parent compound are detected in urine and bile, suggesting limited biliary excretion and negligible enterohepatic circulation (Li et al., 2005).

Manda et al. (2013) further reported that the total degradation rate of DIO in simulated gastric and intestinal fluids was approximately 40%, with part of the compound converted into diosgenin. Nevertheless, substantial amounts of the parent compound remained in the gastrointestinal tract and underwent further metabolism by intestinal microbiota and intestinal epithelial cells (Manda et al., 2013).

Overall, DIO displays a pharmacokinetic profile characterized by low absorption and prolonged intestinal retention. Its disposition is modulated by gut microbiota, CYP450 enzymes and transporter systems, with predominant distribution in the liver and lungs. Unchanged DIO is mainly excreted via feces, whereas urinary and biliary excretion contribute minimally. The comparative pharmacokinetic parameters of DIO are summarized in Table 7.

**TABLE 7:** Pharmacokinetic studies of DIO.

Route of administration	Species	Dose	Pharmacokinetic parameters								References
			T_max_ (h)	C_max_ (ng/mL)	AUC_(0-t)_ (a: ng·h/mL; b: μg·h/mL)	AUC_(0-∞)_ (a: ng·h/mL; b: μg·h/mL)	T_1/2_ (h)	CL (a: mL/min/kg; b: L/h/kg)	MRT_(0-t)_ (h)	Vz (L/kg)	
Oral	Rats (Wistar, male, female)	45 mg/kg	19.4 ± 0.98	9.30 ± 0.91	524 ± 72.8 (a)	536 ± 78.0 (a)	15.2 ± 2.19	1,500 ± 230 (a)	40.1 ± 2.61	2056 ± 103	Li et al. (2005)
Oral	Rats (Wistar, male, female)	90 mg/kg	16.1 ± 3.54	16.5 ± 1.48	951 ± 52.5 (a)	1,002 ± 46.0 (a)	26.6 ± 2.36	1,576 ± 60.7 (a)	47.0 ± 3.07	3,632 ± 400	
i.v	Rats (Wistar, male, female)	0.064 mg/kg	N/A	N/A	254 ± 20.5 (a)	270 ± 9.43 (a)	8.96 ± 1.25	4.67 ± 0.09 (a)	12.5 ± 1.30	3.75 ± 0.30	
i.v	Rats (Wistar, male, female)	0.16 mg/kg	N/A	N/A	678 ± 53.4 (a)	710 ± 64.9 (a)	16.2 ± 2.91	4.45 ± 0.26 (a)	23.0 ± 3.74	5.93 ± 0.75	
i.v	Rats (Wistar, male, female)	0.4 mg/kg	N/A	N/A	1930 ± 231 (a)	1959 ± 221 (a)	21.4 ± 3.54	3.86 ± 0.51 (a)	29.0 ± 5.56	6.76 ± 1.83	
i.v	Rats (Wistar, male, female)	1.0 mg/kg	N/A	N/A	5,095 ± 280 (a)	5,194 ± 283 (a)	22.5 ± 0.83	3.49 ± 0.23 (a)	31.5 ± 1.39	6.60 ± 0.73	
i.v	Rats (SD, male)	3 mg/kg	N/A	N/A	8,976.58 ± 2032.72 (b)	10,344.31 ± 3,318.48 (b)	5.896 ± 1.296	N/A	6.588 ± 0.936	N/A	Zhao et al. (2017)
i.v	Rats (SD, male)	3 mg/kg Soluplus®/TPGS mixed micelles	N/A	N/A	17,719.07 ± 1,540.81 (b)	22,304.59 ± 887.47 (b)	15.22 ± 5.779	N/A	8.465 ± 0.38	N/A	
Oral	Rats (SD, male)	15 mg/kg TSSN (equivalent to 121.8 mg dioscin)	10.29 ± 1.19	38.24 ± 0.63	19,990.91 ± 727.16 (a)	28,379.48 ± 2066.52 (a)	12.79 ± 1.66	0.11 ± 0.01 (b)	25.87 ± 0.74	N/A	Zhang et al. (2015c)
Oral	Rats (SD, male)	20 g/kg crude drug of paridis rhizome	10 ± 2.3	8.61 ± 0.7	219.3 ± 14.9 (a)	227.3 ± 14.6 (a)	14.78 ± 6.1	N/A	23.41 ± 4.1	N/A	Wang et al. (2013)

T_max_, time to maximum plasma concentration; C_max_, maximum plasma concentration; AUC_(0-t)_, area under the concentration-time curve from 0 to t h; AUC_(0-∞)_, area under the concentration-time curve from 0 h to infinity; T_1/2_, elimination half-life; CL, clearance; MRT_(0-t)_, mean residence time from 0 to t h; Vz, clearance, vertical distribution phase; i. v., intravenous administration; Oral, oral administration; N/A, not applicable.

## 10 Drug delivery systems for DIO

### 10.1 Self-assembled nanostructures and liposomal delivery systems

Although DIO possesses diverse pharmacological activities, including antitumor, anti-inflammatory, hepatoprotective and cardioprotective effects, its clinical application is severely restricted by poor aqueous solubility, low oral bioavailability, hemolytic toxicity, tissue irritation, rapid metabolism and insufficient stability *in vivo* (Tao et al., 2018). In recent years, self-assembled nanostructures and liposomal delivery systems have emerged as leading strategies for DIO formulation optimization, effectively improving its solubility, biosafety and targeting capability (Zhang et al., 2024d). Wang et al. (2022) constructed a self-assembled nanostructure based on hydrophobic interactions and hydrogen bonding between DIO and cholesterol, followed by PEGylation modification. This nanoplatform significantly prolonged systemic circulation, reduced hemolytic toxicity, and achieved a 61% tumor growth inhibition rate in a 4T1 breast cancer model, while also avoiding pain caused by free DIO (Wang et al., 2022).

Liu et al. employed sitogluside as a cholesterol substitute to co-assemble with DIO into DPPC-based nanoliposomes. These nanoliposomes displayed markedly enhanced storage stability, gastrointestinal stability, and cellular uptake efficiency (Liu et al., 2024). Compared with free DIO, they exhibited superior hypouricemic and renoprotective effects and effectively suppressed activation of the renal NLRP3 inflammasome pathway in hyperuricemic mice (Liu et al., 2024).

Furthermore, liposomes with multifunctional modifications have attracted considerable attention in combination antitumor therapy. Kong et al. (2020a) developed RPV peptide-modified liposomes co-loaded with DIO and epirubicin, which synergistically enhanced antitumor activity while maintaining a favorable safety profile. Another study reported an MMP2-responsive and CPP-modified liposomal system co-delivering DIO and vinorelbine (Kong et al., 2020b). In this platform, DIO inhibited vasculogenic mimicry, tumor metastasis, and angiogenesis, whereas the liposomal carrier enabled precise tumor targeting and thereby amplified the antitumor efficacy (Kong et al., 2020b). Subsequently, Yao et al. from the same group prepared GGPFV peptide-modified liposomes co-encapsulating daunorubicin and DIO, with an encapsulation efficiency of up to 95.39% (Yao et al., 2020). This formulation markedly enhanced the inhibition of breast cancer cells and effectively overcame the poor solubility and low bioavailability of DIO (Yao et al., 2020).

Apart from combination delivery systems, liposomes containing DIO alone also demonstrated promising therapeutic potential. DIO liposomes prepared using the thin-film hydration method exhibited an average particle size of 125.4 nm and an encapsulation efficiency of 92.7% (Pan et al., 2025). The formulation remained stable after storage at 4 °C for 28 days and showed favorable plasma compatibility, while significantly inhibiting tumor growth in Lewis lung carcinoma-bearing mice (Pan et al., 2025).

### 10.2 Nanosuspensions and mixed micelles

Nanosuspensions and mixed micelles have demonstrated particular promise in improving the solubility and pharmacokinetic behavior of DIO, owing to their relatively simple preparation and low production costs (Meng et al., 2024). A Dio-NS prepared by antisolvent precipitation combined with high-pressure homogenization significantly reduced serum ALT and AST levels, increased SOD activity, decreased MDA accumulation, and alleviated pathological liver injury, thereby exerting pronounced hepatoprotective effects (Ju et al., 2019).

Furthermore, Soluplus®/TPGS mixed micelles loaded with DIO prolonged the mean residence time in blood circulation by 1.3-fold and increased the plasma area under the concentration-time curve by 2.16-fold (Zhao et al., 2017). The micellar formulation also markedly enhanced the *in vitro* antitumor activity against A2780s ovarian cancer cells and MCF-7 breast cancer cells (Zhao et al., 2017).

### 10.3 Stimuli-responsive nanocarriers and nanozyme systems

The development of stimuli-responsive nanocarriers and nanozyme-based systems has further promoted the precise delivery and synergistic therapeutic application of DIO (Wang Y. et al., 2024; Zhang M. R. et al., 2024). For instance, Xu et al. designed a CD11b-modified ROS/pH dual-responsive mesoporous silica nanoparticle system for the co-delivery of DIO and siICAM-1 (Xu et al., 2025). This nanoplatform enhanced targeting efficiency and prolonged systemic circulation. Mechanistically, DIO cooperated with siICAM-1 to reduce neutrophil infiltration by suppressing the NF-κB/STAT3/p38-MAPK signaling pathway, thereby improving cardiac function after myocardial infarction with favorable biosafety profiles (Xu et al., 2025).

Another study constructed a metal-phenolic network-based nanozyme loaded with DIO, glucose oxidase and piceatannol (Ma et al., 2026). Within this multifunctional nanozyme, DIO functioned as the central immunomodulatory agent, driving repolarization of tumor-associated macrophages from the immunosuppressive M2 phenotype to the immunostimulatory M1 phenotype via selective activation of the Cx43-STAT1 signaling axis, thereby reversing the immunosuppressive tumor microenvironment (Ma et al., 2026). This study provides a promising strategy for the application of DIO in cancer immunotherapy.

**FIGURE 3:**
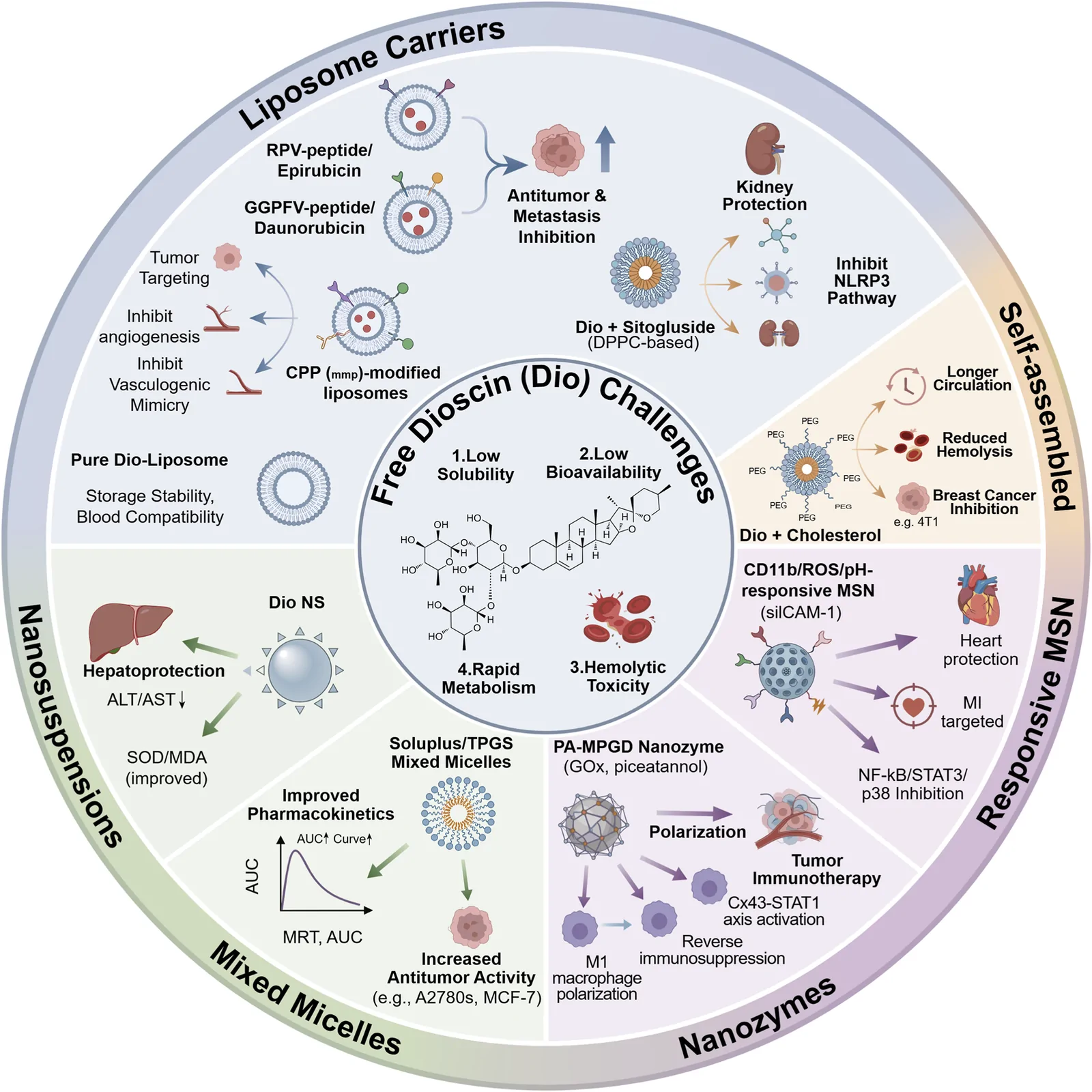
Formulation strategies to improve the solubility, bioavailability and therapeutic efficacy of DIO.

Collectively, a variety of nanotechnology-based delivery systems, including self-assembled nanostructures, liposomes, nanosuspensions, mixed micelles and stimuli-responsive nanoplatforms, have been developed to optimize the pharmaceutical properties of DIO (Figure 3). These novel formulations effectively address the major limitations of DIO, including poor aqueous solubility, low bioavailability, limited targeting efficiency, and potential toxicity. Nevertheless, most current studies remain at the preclinical stage, and challenges related to long-term biosafety, large-scale manufacturing, and clinical translation require further investigation.

## 11 Discussion and future perspective

**FIGURE 4:**
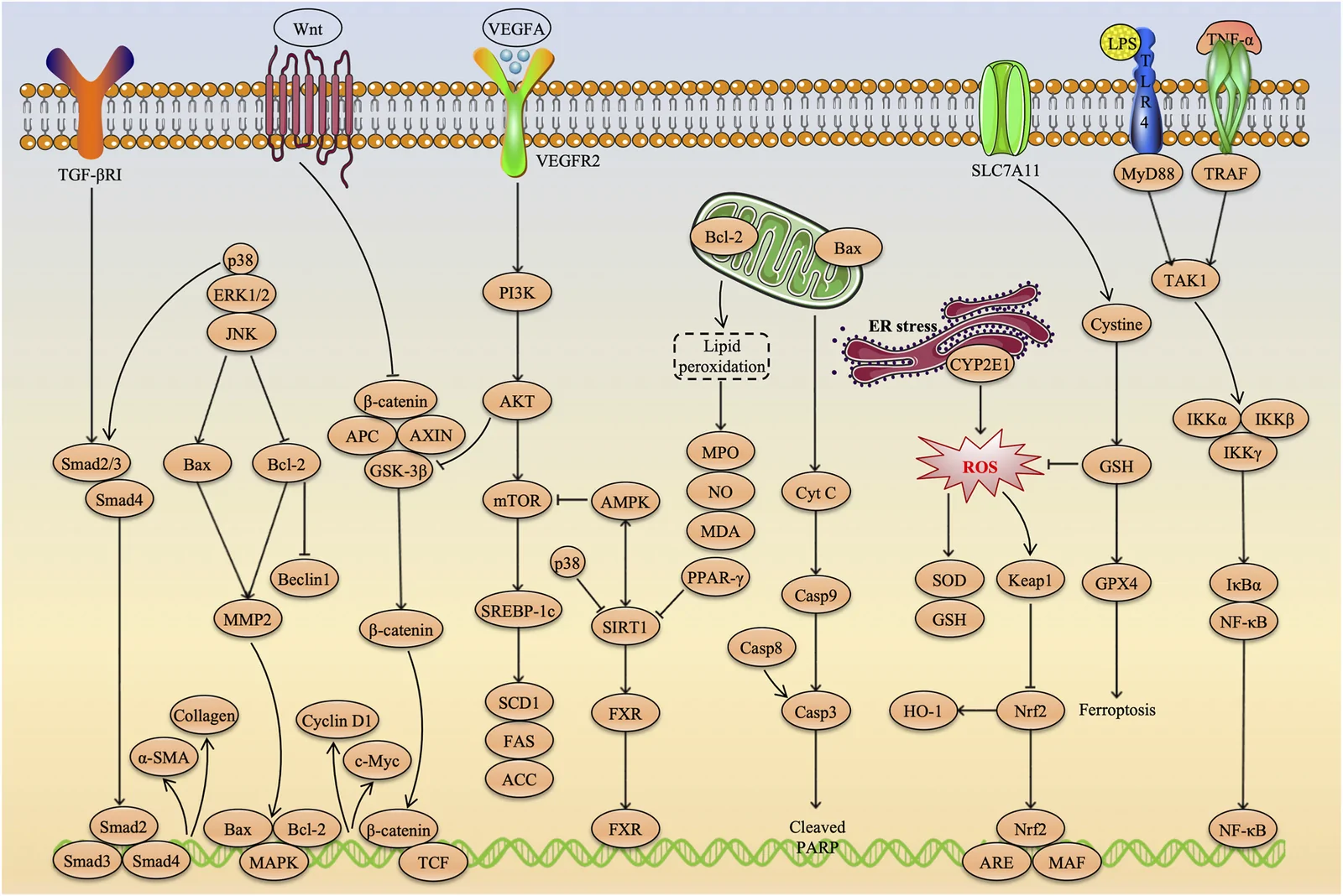
Mechanisms of DIO in the treatment of various liver diseases.

DIO has emerged as one of the most extensively investigated steroidal saponins for the treatment of liver diseases, owing to its broad-spectrum pharmacological activities. A growing body of experimental evidence consistently demonstrates that DIO exerts protective effects against multiple liver disorders, including acute and chronic liver injury, MASLD, cholestatic liver diseases, liver fibrosis and HCC. Rather than acting through a single signaling cascade, DIO coordinately regulates multiple interconnected pathological processes, including oxidative stress, inflammation, lipid metabolism, bile acid homeostasis, programmed cell death and hepatic fibrogenesis (Figure 4). This multitarget pharmacological profile is particularly attractive given the complex pathogenesis of liver diseases, which involves extensive crosstalk among metabolic, inflammatory and immune pathways.

Nevertheless, despite the large number of preclinical studies, current evidence remains largely descriptive and lacks mechanistic integration. Most investigations have relied on rodent models and immortalized cell lines, whereas validation in human tissues, patient-derived organoids and clinical samples is still scarce. Furthermore, published studies exhibit considerable heterogeneity in disease models, dosing regimens, routes of administration and treatment duration, making direct comparisons difficult and limiting reproducibility. More importantly, many studies primarily report alterations in downstream signaling pathways without identifying the initiating molecular events responsible for DIO activity. Consequently, although the hepatoprotective potential of DIO is well supported at the experimental level, the quality of evidence remains insufficient to establish definitive therapeutic mechanisms or predict clinical efficacy.

Another critical limitation concerns the interpretation of the multiple signaling pathways modulated by DIO. Classical pathways such as Keap1/Nrf2, NF-κB, PI3K/Akt, AMPK and TGF-β/Smad are frequently reported not only for DIO but also for many other hepatoprotective phytochemicals, including silymarin, curcumin, resveratrol and berberine. Therefore, activation or inhibition of these pathways may represent common downstream responses to cellular stress rather than DIO-specific mechanisms. The recent identification of ITGA5 and TIGAR as potential molecular targets offers a promising direction for future research and suggests that DIO may exert at least part of its pharmacological activities through selective target engagement. However, these findings remain preliminary and require rigorous biochemical validation. Future studies integrating chemical proteomics, target engagement assays and structural biology will be crucial for distinguishing direct molecular targets from secondary signaling events.

In addition, the functional contribution of these signaling pathways is likely stage-dependent. For instance, modulation of oxidative stress and inflammatory responses appears dominant in early-stage liver injury or MASLD, whereas suppression of hepatic stellate cell activation and extracellular matrix deposition gains prominence during progressive fibrosis. Likewise, modulation of bile acid metabolism holds particular relevance in cholestatic liver diseases, while regulation of tumor metabolism, ferroptosis and the immune microenvironment may contribute more substantially to anti-HCC activity. These observations indicate that DIO functions as a context-dependent network regulator rather than a universal inhibitor of all signaling pathways. Elucidating these spatiotemporal mechanisms is therefore essential for defining precise therapeutic indications and identifying predictive biomarkers to advance precision hepatology.

Equally important but often overlooked is the paradoxical coexistence of hepatoprotective and hepatotoxic effects. Although DIO effectively alleviates liver injury under pathological conditions, several studies have demonstrated that excessive exposure may also induce hepatocellular damage. Rather than representing contradictory findings, these dual effects probably reflect a relatively narrow therapeutic window influenced by dose, exposure duration, disease status and pharmacokinetic properties. At therapeutic concentrations, DIO restores hepatic homeostasis through antioxidant, anti-inflammatory and metabolic regulatory activities. In contrast, high-dose or prolonged administration may trigger oxidative stress, mitochondrial dysfunction, disruption of bile acid transport, dysregulation of CYP450 enzymes and programmed cell death.

Interestingly, these proposed toxicological mechanisms partially overlap with those reported for several clinically recognized hepatotoxic drugs, although important differences remain. For example, bosentan-induced cholestatic liver injury is largely attributed to inhibition of the BSEP, resulting in intrahepatic bile acid accumulation, whereas troglitazone-associated hepatotoxicity is primarily linked to mitochondrial dysfunction, oxidative stress, and the formation of reactive metabolites (Allison et al., 2023; Mahajan et al., 2024). Excessive exposure to DIO has likewise been associated with impaired bile acid homeostasis and mitochondrial injury, suggesting that these processes may represent common pathways underlying drug-induced liver injury. However, unlike bosentan and troglitazone, which are intrinsically associated with hepatotoxicity, DIO exhibits pronounced hepatoprotective activities within an appropriate therapeutic window. Importantly, most toxicological studies have been conducted in healthy animals, whereas pharmacological studies have generally been performed in disease models characterized by oxidative stress and inflammatory activation. Therefore, the balance between efficacy and toxicity is likely to vary substantially according to pathological context, indicating that DIO may exhibit a dose-, exposure-, and disease-dependent dual pharmacological profile rather than an intrinsically hepatotoxic property. These observations underscore the importance of defining exposure-response relationships and therapeutic thresholds through well-designed pharmacokinetic-pharmacodynamic (PK-PD) studies.

The pharmacokinetic properties of DIO further complicate this therapeutic window. Despite an oral bioavailability as low as approximately 0.21%, DIO undergoes extensive first-pass extraction and preferential hepatic distribution after oral administration. Given that the liver serves as both the primary therapeutic target and the major metabolic organ, hepatic accumulation may simultaneously enhance pharmacological efficacy and increase the risk of concentration-dependent toxicity. Disease-associated alterations in hepatic transporters, metabolic enzymes and biliary excretion may further modify intrahepatic drug exposure, suggesting that the therapeutic window of DIO is dynamic rather than fixed. Establishing exposure-response relationships through integrated pharmacokinetic-pharmacodynamic (PK-PD) studies will therefore be essential for optimizing dosage regimens and improving clinical safety.

Poor oral bioavailability represents another major barrier to clinical translation. However, this limitation should not be viewed simply as a consequence of poor aqueous solubility. Instead, it results from multiple factors, including limited intestinal absorption, extensive first-pass metabolism and, importantly, microbiota-mediated biotransformation. Following oral administration, DIO is extensively deglycosylated by intestinal microorganisms to produce diosgenin and other metabolites before systemic absorption. This raises a critical question that has not yet been adequately addressed: Are the pharmacological effects primarily mediated by the parent compound or by its microbial metabolites? Although diosgenin itself has demonstrated anti-inflammatory and anti-fibrotic activities in experimental models, comparative studies directly evaluating the respective contributions of DIO and its metabolites remain scarce. Clarifying this issue is essential because it will fundamentally influence future drug development strategies.

This unresolved question also has important implications for pharmaceutical formulation. Numerous nanotechnology-based delivery systems, including liposomes, polymeric nanoparticles, mixed micelles and self-assembled nanostructures, have been developed to improve the solubility and systemic exposure of DIO. Nevertheless, whether these formulations truly overcome the major pharmacokinetic bottleneck remains uncertain. If microbial metabolism is required to generate active metabolites, excessive protection of DIO from intestinal degradation could theoretically reduce therapeutic efficacy. Conversely, if the parent compound is the principal active species, minimizing microbial metabolism would be advantageous. Therefore, future formulation strategies should not merely aim to increase bioavailability but should instead be guided by a comprehensive understanding of microbiota-drug interactions, active metabolite formation and tissue-specific pharmacokinetics.

Although considerable progress has been made in elucidating the pharmacology of DIO, substantial challenges remain before successful clinical translation can be achieved. Firstly, standardized manufacturing processes and quality control systems are required to minimize batch-to-batch variability associated with botanical sources and extraction procedures. Secondly, comprehensive toxicological evaluations, including long-term toxicity, reproductive toxicity, genotoxicity and potential drug-drug interactions, are essential because patients with chronic liver diseases frequently receive multiple concomitant medications. Thirdly, regulatory approval will require robust evidence regarding pharmacokinetics, target engagement, mechanism of action and safety, all of which remain incompletely characterized for DIO.

Clinical trial design also warrants careful consideration. Given the heterogeneity of liver diseases, future studies should focus on clearly defined patient populations and disease stages rather than attempting to evaluate DIO across all liver disorders simultaneously. In MASLD, endpoints related to metabolic improvement and histological resolution may be appropriate, whereas fibrosis regression and non-invasive fibrosis biomarkers may be more informative in advanced liver fibrosis. For HCC, DIO is more likely to serve as an adjuvant therapy combined with existing systemic treatments than as a stand-alone anticancer agent. Moreover, interindividual differences in gut microbiota composition, hepatic metabolism and immune responses may substantially influence therapeutic efficacy, highlighting the importance of integrating biomarkers, multi-omics technologies and precision medicine approaches into future clinical studies.

In summary, DIO represents a highly promising natural compound with multitarget pharmacological activities and broad therapeutic potential for liver diseases. However, However, successful clinical translation will require moving beyond descriptive pathway analyses to identify direct molecular targets, clarify active metabolites, define the therapeutic window, and resolve pharmacokinetic bottlenecks. Future investigations should integrate systems pharmacology, multi-omics technologies, PK-PD modeling and well-designed clinical trials to establish a robust evidence base for DIO. Addressing these critical challenges will not only facilitate the development of DIO as a novel therapeutic agent but also provide valuable insights into the rational development of multitarget natural products for complex liver diseases.

## Glossary

AKT: protein kinase B
ALP: alkaline phosphatase
ALT: alanine aminotransferase
AMPK: AMP-activated protein kinase
ANIT: α-naphthylisothiocyanate
APAP: acetaminophen
AST: aspartate aminotransferase
BSEP: bile salt export pump
BDL: bile duct ligation
**CCl**_**4**_: carbon tetrachloride
Col1a1: collagen type I alpha 1
DIO: dioscin
DIO-NS: DIO nanosuspension
DMN: dimethylnitrosamine
DOX: doxorubicin
ECM: extracellular matrix
FAK: focal adhesion kinase
EMT: epithelial-mesenchymal transition
FGF21: fibroblast growth factor 21
GLP-1: glucagon-like peptide-1
GSH: glutathione
GSH-Px: glutathione peroxidase
GST: glutathione S-transferase
HCC: hepatocellular carcinoma
HFD: high-fat diet
HO-1: heme oxygenase 1
**HC**_**50**_: half-hemolytic concentration
HSC: hepatic stellate cell
HYP: hydroxyproline
IL-1β/6/10: interleukin-1β/6/10
iTRAQ: isobaric tags for relative and absolute quantitation
LDH: lactate dehydrogenase
LPS: lipopolysaccharide
MASLD: metabolic dysfunction-associated steatotic liver disease
MDA: malondialdehyde
MDR: multidrug resistance
mTOR: mammalian target of rapamycin
MTX: methotrexate
MRP2: multidrug resistance-associated protein 2
OATPs: organic anion transporting polypeptides
NF-κB: nuclear factor kappa
Nrf2: nuclear factor erythroid 2-related factor 2
P-gp: P-glycoprotein
PI3K: phosphatidylinositide 3-kinase
PKCα: protein kinase Cα
PPARα: peroxisome proliferator-activated receptor alpha
ROS: reactive oxygen species
SHP: small heterodimer partner
SDC-4: Syndecan-4
SIRS: systemic inflammatory response syndrome
SOD: superoxide dismutase
STAT3: signal transducer and activator of transcription 3
TAA: thioacetamide
TCM: traditional Chinese medicine
TGF-β1: transforming growth factor β1
TIGAR: TP53-induced glycolysis and apoptosis regulator
TNF-α: tumor necrosis factor α
TCM: traditional Chinese medicine
α-SMA: α-smooth muscle actin
PK-PD: pharmacokinetic-pharmacodynamic.
